# Environmental impact of urine analyses: Comparison of manual, semi and fully automated analytical workflows

**DOI:** 10.1016/j.joclim.2026.100737

**Published:** 2026-09-10

**Authors:** Julien Brunier, Clara Mourgues, Fanny Auger, Gérard Lina, François Vandenesch, Céline Ramanantsoa, Olivier Dauwalder

**Affiliations:** a Centre Hospitalier Le Mans, Laboratoire de microbiologie, Le Mans, France; b EHESP - École des Hautes Études en Santé Publique, 15 Av. du Professeur Léon Bernard, 35043 Rennes, France; c Primum non nocere, Béziers, France; d Hospices Civils de Lyon, 24/7 Microbiology Platform, Institut des Agents Infectieux, Centre de Biologie et Pathologie Nord, Lyon, France; e CIRI, Centre International de Recherche en Infectiologie, Lyon University, Inserm, U1111, University Claude Bernard Lyon 1, CNRS, UMR5308, ENS de Lyon, Lyon, France

**Keywords:** Urine analysis, Carbon footprint, Life cycle assessment, Healthcare ecodesign, Microbiological laboratory automation, Antibiotic susceptibility testing

## Abstract

**Objectives:**

Urinary tract infection is the most common infection requiring urinalysis (UA). Due to economical constraints, many laboratories have merged to use full laboratory automation instruments for high volume of urine samples. However, the environmental impact (EI) of UA is still unknown, particularly according to automation levels. The present study aimed to document the environmental impact of UA for the first time.

**Methods:**

We used the life cycle assessment (LCA) methodology (ISO 14040/44, 2006) to compare manual, semi-automated and fully-automated analytical workflows for *Escherichia coli* UA (ECUA) including cytology, isolation, and antibiotic susceptibility tests (AST). Using a multicenter approach, we measured midpoints and endpoints levels defined by the impact assessment method ReCiPe 2016.

**Results:**

ECUA carbon footprint was 1.9, 2.6 and 1.1 kgCO2eq using manual, semi-automated and fully-automated workflows respectively. AST processes accounted for 66.8%, 79.0%, and 59.9% of emissions, respectively. The fully-automated workflow had the lowest impact for 12/18 midpoint indicators and had the lowest impact on human health (2.9 × 10–6 disability-adjusted life years (DALY) vs 3.7 × 10–6 for manual and 6.9 × 10–6 for semi-automated workflows). When scaled to 50,000 ECUA/year, the manual workflow had the lowest impact for 10/18 midpoints and human health (3.7 × 10–6 DALY), but its impact remained higher than fully-automated in real conditions.

**Conclusions:**

The EI of UA depends on both the automation level and the organizational scale. While automation does not always reduce impacts linearly, full automation offers the best carbon and health performance in high-throughput settings. Integrating LCA into laboratory strategy may support the ecological transition of microbiology.

## Highlights

• Automating urinalysis reduces carbon emissions and health-related impacts.
• Antibiotic susceptibility test step generates most of urinalysis environmental footprint.
• Urinalysis emits as much CO_2_ as driving 11 to 27 km by car, depending on the workflow.
• Lab consumables have high impact but offer clear opportunities for reduction.
• Eco-design and diagnostic stewardship can significantly lower lab environmental burden.

## Graphical abstract

**Figure:**
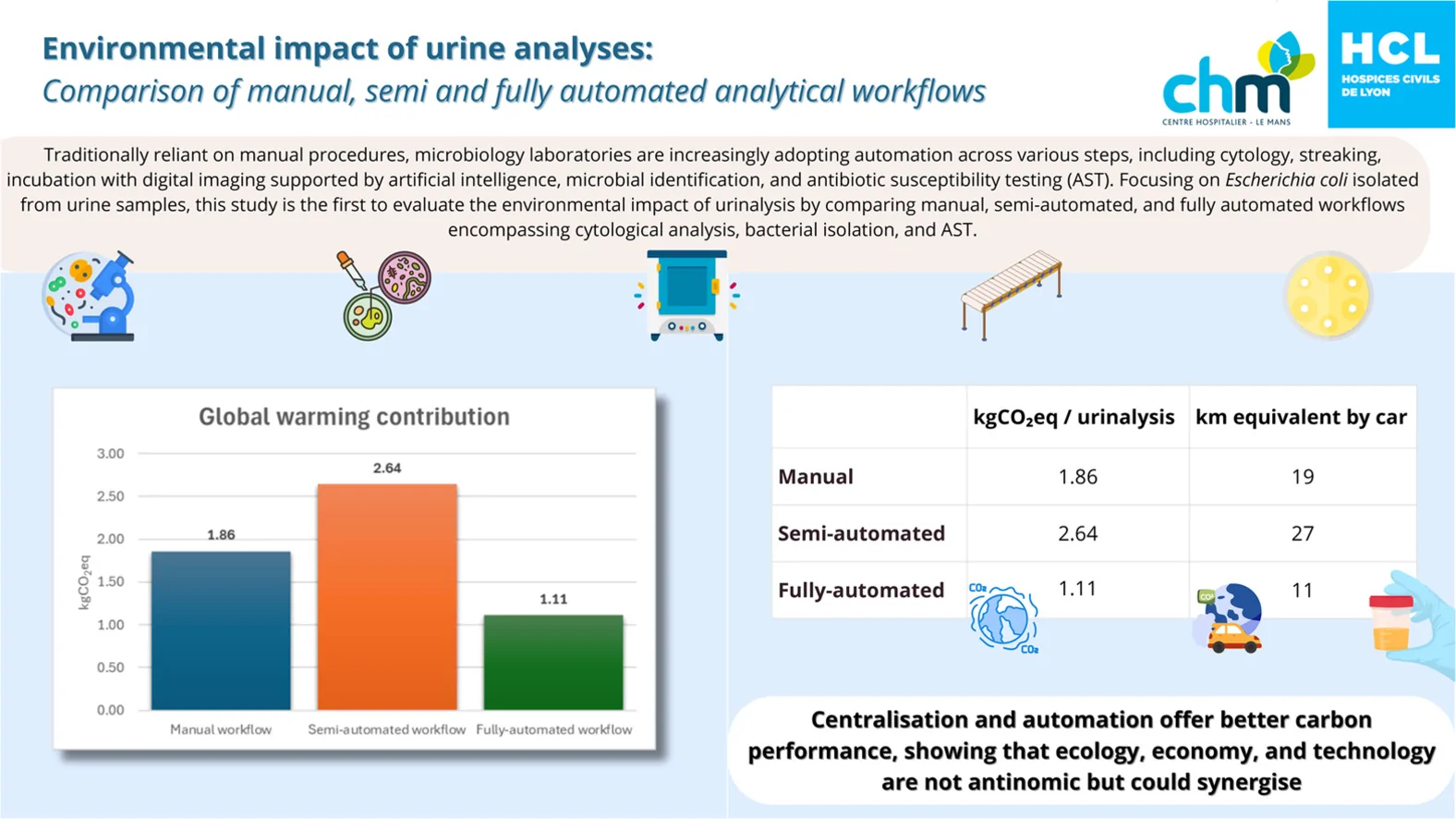

## 1 Introduction

Healthcare delivery is increasingly recognized as a major contributor to environmental degradation [1]. The healthcare sector accounts for approximately 4.4% of global greenhouse gas (GHG) emissions [2] and nearly 8% of national emissions in France [3]. These emissions are largely driven by energy and material consumption across care pathways, while diagnostic activities remain comparatively underexplored [4].

Climate change, one of the nine planetary boundaries defining a safe operating space for humanity, is among the seven already exceeded [5], raising concerns about the long-term sustainability of healthcare systems. Because GHG emissions are closely linked to fossil fuel use, healthcare systems must rapidly reduce fossil fuel consumption in line with the Paris Agreement to mitigate associated health impacts [6,7]. However, increasing energy scarcity, including “peak oil” [8], and dependence on imported fuels threaten the resilience of healthcare infrastructures, particularly in countries such as France [9]. Healthcare systems are therefore confronted with a double constraint: reducing their contribution to climate change while maintaining the continuity of care under growing energy constraints. This situation also raises ethical concerns, as healthcare systems are both contributors to climate change and responsible for protecting population health [10]. These pressures are compounded by the direct impacts of environmental degradation on healthcare facilities and supply chains [11].

In parallel, regulatory frameworks are evolving rapidly. European Environmental, Social and Governance regulations, including the Corporate Sustainability Reporting Directive and the Corporate Sustainability Due Diligence Directive, are progressively extending sustainability requirements to healthcare organizations and clinical laboratories [12,13]. Addressing these challenges requires integrating environmental indicators into performance management and conducting comprehensive assessments of healthcare processes. To date, however, such detailed assessments remain scarce for routine microbiology diagnostic tests at the level of analytical workflows.

Against this backdrop, urinalysis represents a particularly relevant case study. Diagnosis of urinary tract infection (UTI) has historically relied on urinalysis (UA) [14,15] combining cytology and conventional culture. UA is among the most frequently prescribed medical analyses in France, accounting for 11 million tests in 2023 [16]. Recent advances allow full automation through integrated cytology instruments, plate streakers, digital incubators with artificial intelligence, and automated systems for colony picking and antibiotic susceptibility testing (AST) preparation [17]. As a high-volume, standardized diagnostic activity undergoing increasing automation and organizational centralization, UA raises important but insufficiently documented questions regarding the environmental implications of technological complexity and workflow organization. This study therefore assesses the environmental impact of *Escherichia coli*urinalysis (ECUA) in France across manual, semi-automated, and fully automated workflows using life cycle assessment methodology, and identifies potential optimization pathways.

## 2 Material and methods

### 2.1 Study setting

The study was conducted in two hospital microbiology laboratories: Le Mans hospital for manual and semi-automated workflows, and the Lyon university hospital for a fully-automated workflow, processing 41,000 and 130,000 samples annually, respectively.

### 2.2 Study design

A hybrid life cycle assessment (LCA) combining process-based and input-output methods was conducted in accordance with ISO 14,040/44 [18].

Because *E.coli* is the main cause of UTI worldwide [19], we defined the functional unit as the analytical phase of one ECUA. The functional unit represents the quantified function delivered by the laboratory process, a reference to compare environmental impacts between workflows. It focuses on the diagnostic service provided rather than on the individual components used to perform the test. To ensure comparability across settings, the scope was limited to the analytical phase, common to both hospital and outpatient laboratories. This phase included registration, cytology, direct examination, plate inoculation/streaking, incubation, antibiotic susceptibility test (AST) preparation, plate reading, and technical interpretation.

Allocation was required to distribute the environmental impact of shared instruments and resources to the functional unit, as the same instruments and consumables are often used for other analyses. Two allocation scenarios were therefore applied: one reflecting real-life consumables and instruments use (real-life rule), and one normalizing impacts over 50,000 ECUA per year (theoretical scale smoothing rule).

The smoothing approach was used to model the three technological workflows at a common activity level in order to isolate the effect of technology from the effect of laboratory scale observed in real-life settings. The value of 50,000 ECUA was selected as an intermediate activity level between the two study sites and was used as an illustrative scenario to explore scale effects rather than to define an optimal threshold.

### 2.3 Comparison of three analytical workflows

The functional unit was evaluated according to three workflows, each corresponding to a separate life cycle. Manual: manual plate streaking of chromogenic media, manual reading, microscopic cytology, and disc diffusion AST according to CASFM/EUCAST rules [20]; semi-automated: automated plate streaking, manual reading of chromogenic media, microscopic cytology, and semi-automated AST according to CASFM/EUCAST rules [20]; fully-automated: automated cytology, laboratory automation system (streaking instrument, conveyors and incubators for chromogenic media, automated reading using artificial intelligence software), and automated AST according to CASFM/EUCAST rules [20]. Workflow inventories are detailed in Supplementary Tables 1–3.

### 2.4 Indicators

We used the ReCipe 2016 impact assessment method [21], illustrated in Supplementary Figure 1, to evaluate both direct and indirect environmental impacts. This method includes indicators at two levels: “midpoint” and “endpoint”. Midpoint indicators represent specific environmental factors along the cause-impact pathway, such as global warming, acidification, or freshwater eutrophication, and are generally more robust and reliable for environmental interpretation. Endpoint indicators reflect aggregated damage to three areas of protection: human health, ecosystem quality, and resource scarcity. However, endpoint calculations rely on modeling assumptions that can introduce significant uncertainty, particularly for resource-related or economic interpretations. We nevertheless reported the human health endpoint, expressed in disability-adjusted life years (DALY), to provide a health-oriented perspective alongside environmental indicators. This dual-level approach provides a comprehensive assessment of environmental impacts, aligning with internationally recognized life cycle assessment practices.

### 2.5 Data collection and processing

#### 2.5.1 Inventory

**Fig. 1:**
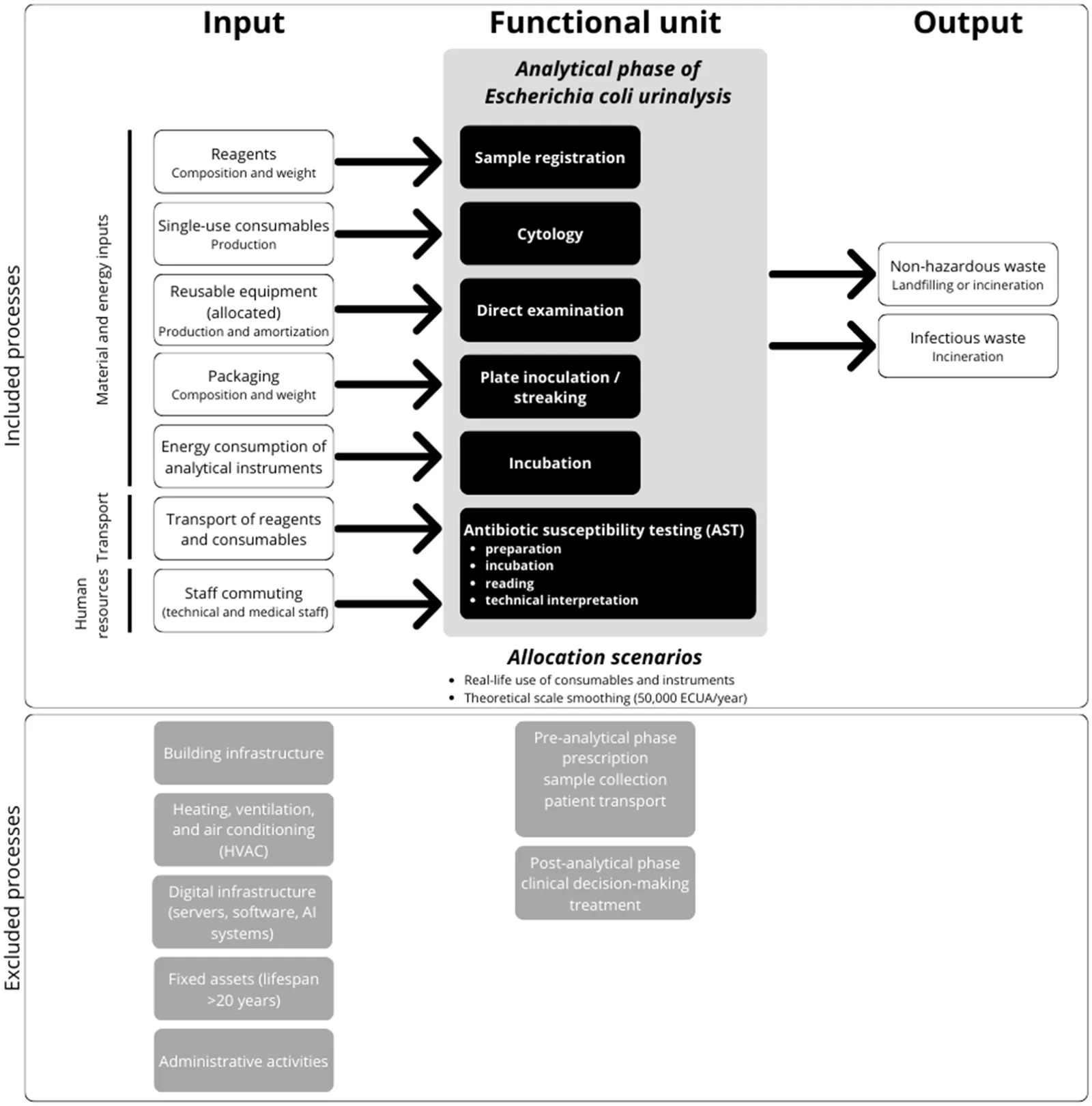
System boundary diagram of the life cycle assessment of *Escherichia coli*urinalysis (ECUA). The diagram shows the processes included in the analytical phase of one ECUA and those excluded from the system boundary.

Data were collected in 2022 from product sheets and safety data sheets, and material weights were measured using a precision balance (PCE-BSK 310®, PCE Instruments, Soultz-sous-Forêts, France). A plastics engineer provided the missing composition data. Transport distances were estimated via www.searates.com, using production sites or supplier headquarters when unavailable. Consumables and instruments with negligible characterization factors (e.g. labels or paper) or lifespans exceeding 20 years (e.g. disc distributor) were excluded. Fixed assets and building infrastructure impacts were excluded due to data unavailability and Le Mans' building age (>30 years). Energy for heating, cooling, and air conditioning was omitted due to lack of metering (Fig. 1).

Daily ECUA volumes were extracted from laboratory information systems: InLog software (Limonest, France) for Le Mans and GLIMS 10 (Clinisys, Ghent, Belgium) for Lyon. Waste treatment data were obtained through interviews with sector engineers.

#### 2.5.2 Commuting documentation

A mobility survey was conducted using an anonymous questionnaire among the technical and biological staff at both sites. The results of this survey were used to determine the modal split (proportion of each mode of transport) and allocated it with the following assumptions: 2 technical full-time equivalents (FTEs) and 1 medical FTE for manual workflow; 1.5 technical FTEs and 1 medical FTE for semi-automated workflow; and 1 technical FTE and 1 medical FTE for fully-automated workflow.

#### 2.5.3 Modeling

Environmental impact modeling was conducted using the Ecoinvent v3.0 database (Swiss Centre for Life Cycle Inventories, Zurich, Switzerland) and SimaPro software (PRé Consultants, Amersfoort, Netherlands). The impact of ECUA workflows was calculated by multiplying the physical life cycle data of each component (quantity, material composition, packaging, energy consumption, transport, end-of-life) by their respective characterization factors, which indicate the impact per unit of material or activity. Impacts were classified in each midpoint category and then aggregated into endpoints. The uncertainty associated with input-output data is approximately 20% and therefore applies to all results in this study.

To facilitate interpretation, results for the global warming indicator (carbon footprint), the most commonly reported environmental indicator, are also expressed using an illustrative equivalent: the emissions of an average thermal (internal combustion) car emitting 0.097 kgCO_2_eq per km [22]. These values were then re-scaled to the national level using the mean *E. coli* positivity rate of UA observed at the two study sites, 23% (19% in Le Mans and 27% in Lyon). Based on this rate, it was estimated that at least 2,474,473 ECUA are performed annually out of the 10,758,580 UA conducted each year in France [16].

No patient data were collected; staff data, obtained through surveys and anonymous questionnaires, complied with the General Data Protection Regulation (GDPR). According to national legislation in place at the time of the study in France, ethics committee approval was not required for this study.

## 3 Results

### 3.1 Midpoint indicator results

The fully-automated workflow had the lowest impact for 12/18 (66.7%) midpoints as compared to the manual and semi-automated workflows, and the manual workflow had the lowest impact for 6/18 (33.3%). The fully-automated workflow’s carbon footprint (kgCO_2_eq) was 1.7 times lower than manual and 2.4 times lower than semi-automated (Table 1). Using a theoretical scale smoothing rule standardized at 50,000 ECUA, the manual workflow had the lowest impact for 10/18 (55.6%) midpoints, and the fully-automated workflow had the lowest impact for 8/18 (44.4%). The fully-automated workflow’s carbon footprint was 1.3 times lower than manual and 1.7 times lower than semi-automated (Supplementary Table 4).

**Table 1:** Urinary quantitative environmental impacts (midpoints) and quantitative damage at three key areas of protection (endpoints) for one *Escherichia coli* urinalysis in the three workflows in real-life rule, according to ReCiPe2016.

	Unit	manual workflow	semi-automated workflow	fully-automated workflow
Midpoint				
Global warming	kgCO_2_eq	1.858	2.643	**1.114**
Stratospheric ozone depletion	kgCFC11eq	7.5 × 10^–7^	11.5 × 10^–7^	**5.3×10^–7^**
Ionising radiation	kBqCo-60eq	**0.303**	0.442	0.825
Ozone formation, Human health	kgNOxeq	0.004	0.006	**0.003**
Fine particulate matter formation	kgPM2.5eq	0.002	0.003	**0.001**
Ozone formation, terrestrial eco.	kgNOxeq	0.004	0.006	**0.003**
Terrestrial acidification	kgSO2eq	0.005	0.008	**0.003**
Freshwater eutrophication	kgPeq	6.3 × 10^–4^	10.0 × 10^–4^	**5.8×10^–4^**
Marine eutrophication	kgNeq	**3.7** × **10^–5^**	6.7 × 10^–5^	6.9 × 10^–5^
Terrestrial ecotoxicity	kg1,4-DCB	6.172	7.019	**4.994**
Freshwater ecotoxicity	kg1,4-DCB	**0.102**	0.442	0.182
Marine ecotoxicity	kg1,4-DCB	**0.135**	0.582	0.237
Human carcinogenic toxicity	kg1,4-DCB	0.137	0.200	**0.091**
Human non-carcinogenic toxicity	kg1,4-DCB	**1.616**	6.973	2.621
Land use	m^2^acropeq	0.084	0.090	**0.070**
Mineral resource scarcity	kgCueq	**0.005**	0.024	0.009
Fossil resource scarcity	kgoileq	0.522	0.675	**0.353**
Water consumption	m^3^	0.014	0.019	**0.013**
Endpoints				
Human health	DALY	3.7 × 10^–6^	6.9 × 10^–6^	**2.9×10^–6^**
Ecosystems	species.yr	8.2 × 10^–9^	12.1 × 10^–9^	**5.5×10^–9^**
Resources	USD2013	0.203	0.221	**0.125**

Units are expressed in terms of kilograms (kg) per equivalent (eq) of carbon dioxide to air (CO_2_), of trichlorofluoromethane to air (CFC11), of cobalt-60 to air (Co-60), of nitrogen oxides to air (NOx), of particulate matter of 2.5 ^µm^or smaller to air (PM2.5), of sulphur dioxide to air (SO_2_), of potassium to freshwater (P), of nitrogen to marine water (N) of 1,4-dichlorobenzene to industrial soil, to freshwater, to marine water, to urban air (1,4-DCB); in terms of square meters of annual cropland (m^2^acrop); in terms of kilograms per equivalent of copper (Cu), of oil; in terms of cubic meters of water (m^3^), in terms of disability-adjusted life years (DALY); in terms of number of species lost (species.yr); in terms of surplus costs of future resource production (USD2013). The lowest impact between the three workflows is indicated in bold.

### 3.2 Endpoint indicator results

The fully-automated workflow had the lowest impact for the three indicators in real-life rule (Table 1). The impact of carrying out an ECUA on human health was 3.7 × 10^–6^ DALY for the manual workflow, 6.9 × 10^–6^ DALY for the semi-automated workflow, and 2.9 × 10^–6^ DALY for the fully-automated workflow. Using a theoretical scale smoothing rule standardized at 50 000 ECUA, the manual workflow had the lowest impact for human health and the fully-automated workflow had the lowest impact on ecosystems and resources area. The impact of carrying out an ECUA on human health was 3.7 × 10^–6^ DALY for the manual workflow, 5.8 × 10^–6^ DALY for the semi-automated workflow, and 4.4 × 10^–6^ DALY for the fully-automated workflow (Supplementary Table 4).

### 3.3 Environmental impact of consumables and instruments

**Fig. 2:**
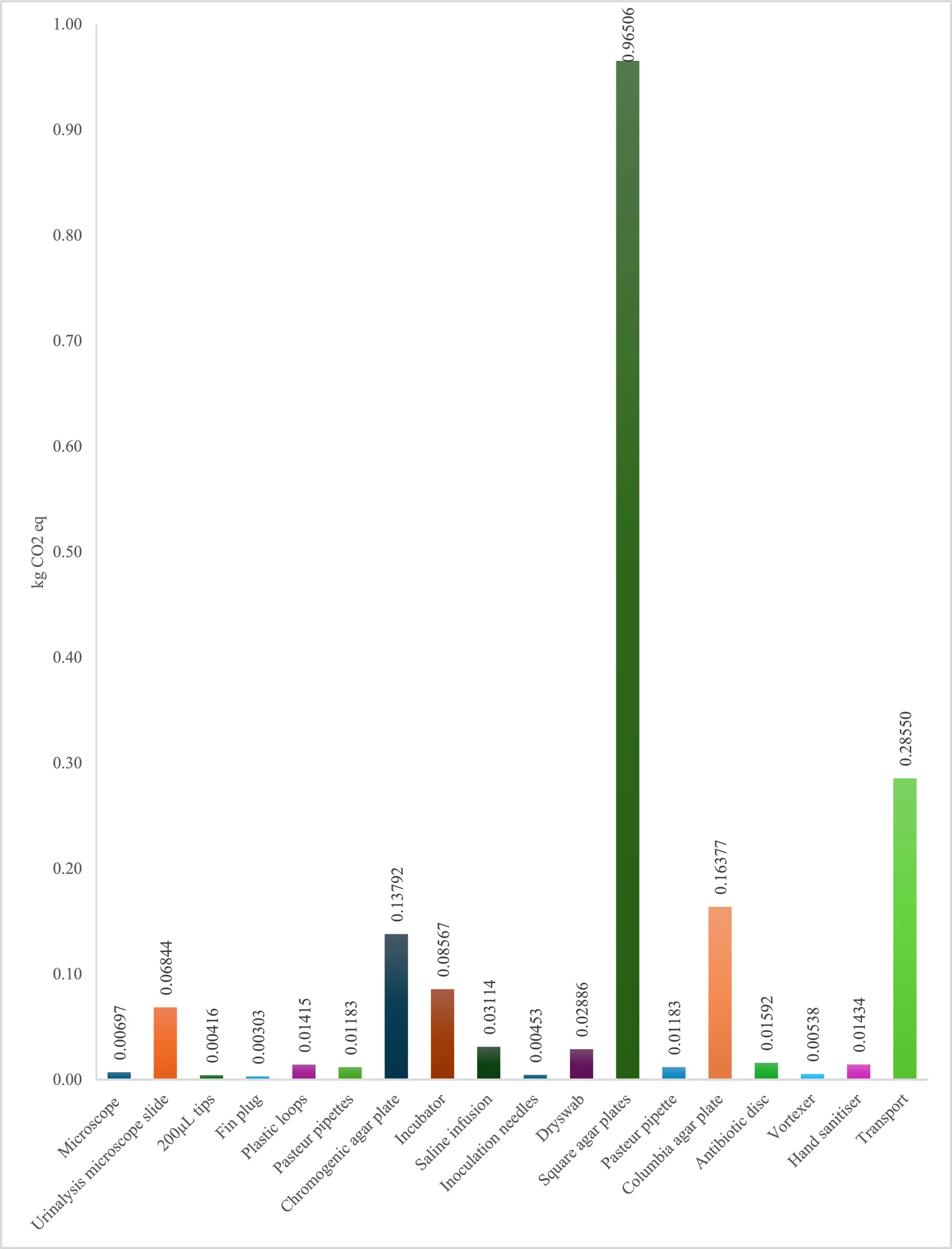
Carbon footprint of individual components of the manual workflow in real-life at Le Mans laboratory. Climate change impacts are expressed in kilograms of CO₂ equivalent (kgCO₂eq) for each component of the workflow, highlighting the relative contribution of consumables, reagents, instruments, and human resources to the overall carbon footprint.

**Fig. 3:**
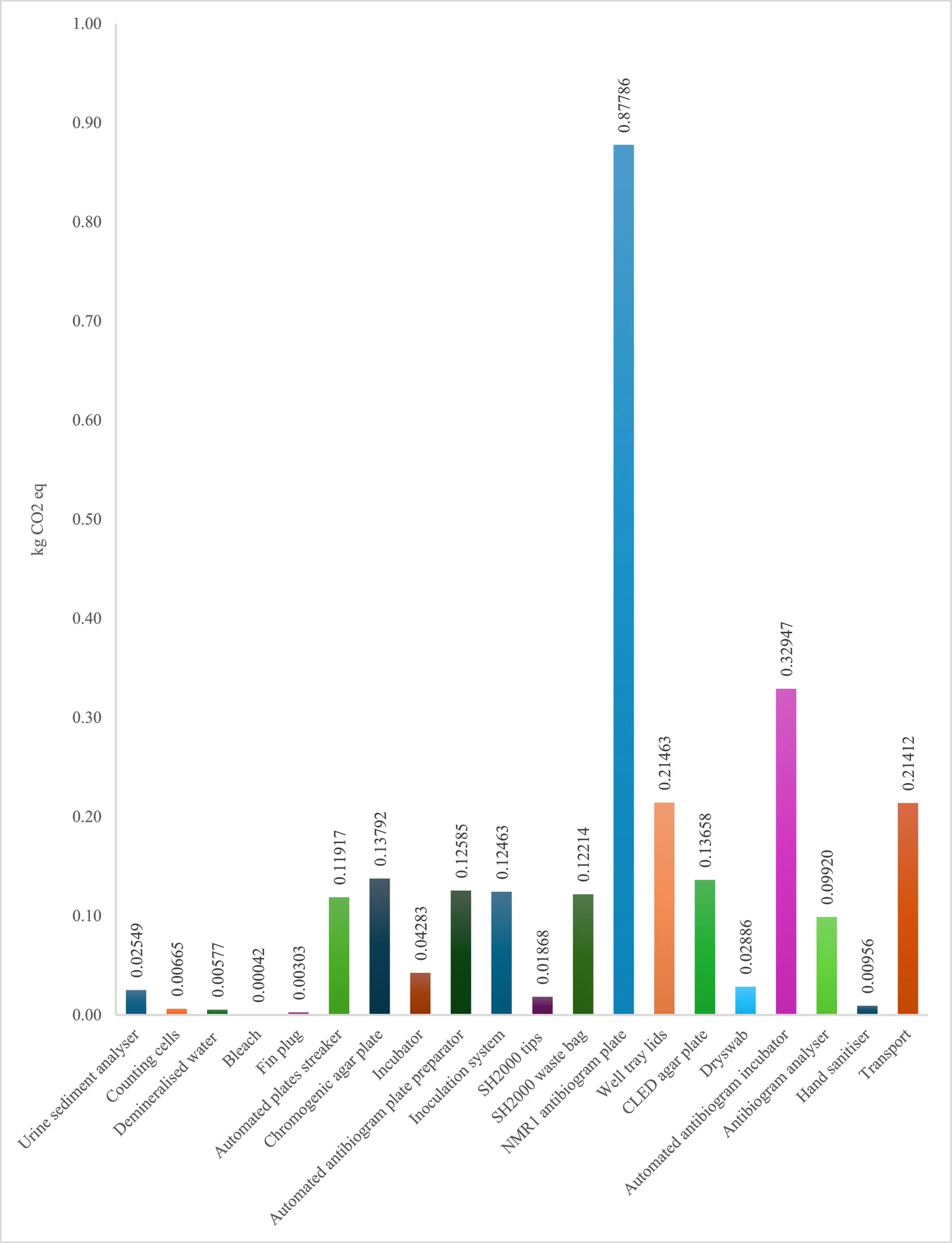
Relative environmental impacts of semi-automated workflow in real-life at Le Mans laboratory is analysed using the 18 indicators of Product Environmental Footprint method and distributed between reagents, consumables, instruments and human resources used.

**Fig. 4:**
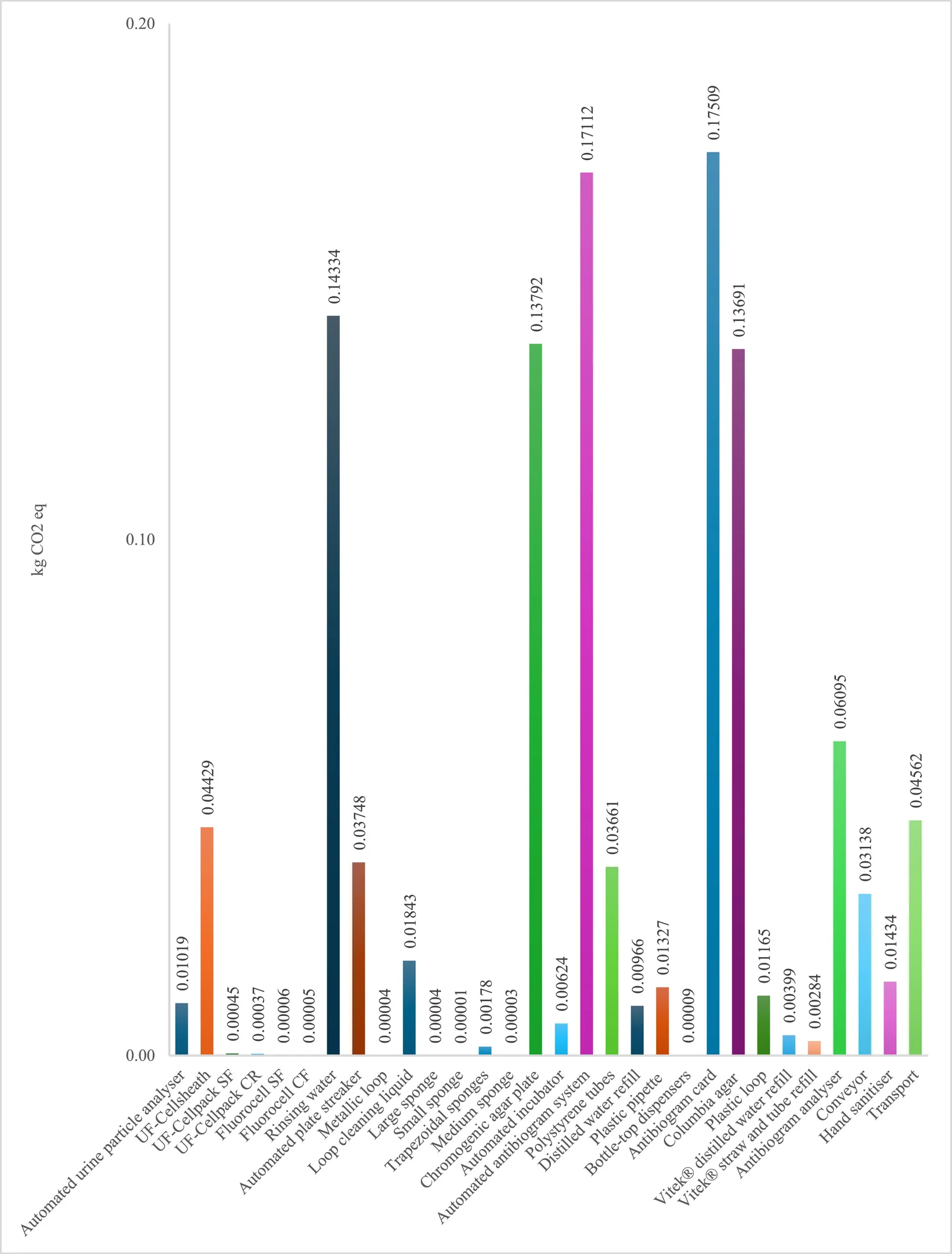
Carbon footprint of individual components of the fully-automated workflow in real-life at Lyon laboratory. Climate change impacts are expressed in kilograms of CO₂ equivalent (kgCO₂eq) for each component of the workflow, highlighting the relative contribution of consumables, reagents, instruments, and human resources to the overall carbon footprint.

In the manual workflow, the square agar plates (51.9%) contributed the most to carbon footprint, followed by Columbia agar plate (8.8%), chromogenic agar plate (7.4%), and the incubator (4.6%; Fig. 2). In the semi-automated workflow, antibiogram plate (33.2%) contributed the most to carbon footprint, followed by the automated antibiogram incubator (12.5%; Fig. 3). In the fully-automated workflow, the antibiogram card (15.7%) and the automated antibiogram system (15.4%) contributed the most to carbon footprint, followed by bottled water used for rinsing in cytology (12.9%) and chromogenic agar plate (12.4%; Fig. 4). Details on consumables and instruments are in Supplementary Figures 2–7. The assessment of other environmental impacts of consumables and instruments for each workflow is presented in Supplementary Figures 8–10.

### 3.4 Environmental impact of sub-process

In manual workflow, cytology accounted for 4.4% of carbon footprint (8.0 × 10^–2^ kgCO₂eq), standard bacteriology (streaking and incubation) for 13.4% (0.3 kgCO₂eq) and AST processes (from preparation to reading) for 66.8% (1.2 kgCO₂eq) (Fig. 2). In semi-automated workflow, cytology accounted for 1.6% of carbon footprint (4.0 × 10^–2^ kgCO₂eq), standard bacteriology for 11.4% (0.3 kgCO₂eq) and AST processes for 79.0% (2.1 kgCO_2_eq) (Fig. 3). In fully-automated workflow, cytology accounted for 17.8% of carbon footprint (0.2 kgCO₂eq), standard bacteriology for 18.1% (0.2 kgCO₂eq) and AST processes for 59.9% (0.7 kgCO_2_eq) (Fig. 4).

Transportation of technical staff represented the second largest source of carbon emissions in the manual workflow (15.4% of total emissions), the fourth in the semi-automated workflow (8.1%), and the seventh in the fully automated workflow (4.1%) (Fig. 2, Fig. 3, Fig. 4).

### 3.5 Results in CO_2_ and kilometers per car equivalent

Per ECUA, GHG emissions were to 1.9 kgCO₂eq for the manual workflow, 2.6 kgCO₂eq for the semi-automated workflow, and 1.1 kgCO₂eq for the fully-automated workflow (Table 1). This was equivalent to, respectively 19.2 km, 27.2 km and 11.4 km driven by an average thermal car.

When extrapolated to annual local activity, these emissions represent 92,168 km (approximately 2.3 trips around the Earth) for the manual workflow (Le Mans), 130,406 km (3.3 trips around the Earth) for the semi-automated workflow (Le Mans), and 223,351 km (5.6 trips around the Earth) for the fully-automated workflow (Lyon).

Scaled to national activity, this corresponds, respectively for manual, semi-automated and fully-automated workflow, to 4,602,520 kgCO_2_eq, 6,540,033 kgCO_2_eq and 2,756,563 kgCO_2_eq, equivalent to 1,208, 1,715, and 721 trips around the Earth (Table 2). It is equivalent to 7.4 × 10^–3^, 10.5 × 10^–3^ and 4.4 × 10^–3^ of the French national carbon footprint in 2022 [23].

**Table 2:** Global warming impact expressed in terms of carbon footprint and kilometres per car equivalent of the Le Mans laboratory, the Lyon laboratory, and nationally for *Escherichia coli*urinalysis activity in 2022.

		Le Mans, ECUA, n = 4,786	Lyon ECUA, n = 19,448	National ECUA, n = 2,474,473
Manual	Carbon footprint, kgCO_2_eq	8,940	36,173	4,602,521
	Distance equivalent, km	92,168	372,920	47,448,665
Semi-automatic	Carbon footprint, kgCO_2_eq	12,649	51,401	6,540,033
	Distance equivalent, km	130,406	529,908	67,423,023
Automatic	Carbon footprint, kgCO_2_eq	5,332	21,665	2,756,563
	Distance equivalent, km	54,965	223,351	28,418,179

Results are expressed in terms of the emissions of an average car emitting 0.097 kgCO_2_eq per kilometre (km) [22], re-scaled to the national level using the mean *E. coli*positivity rate of urine analysis observed at the two study sites, 23% (19% in Le Mans and 27% in Lyon). Units are expressed in terms of kilograms (kg) per equivalent (eq) of carbon dioxide to air (CO_2_) or per equivalent kilometres (km).

## 4 Discussion

### 4.1 Key findings

In the present study, the fully-automated workflow had the lowest environmental impact in terms of global warming and the lowest impact on human health under the real-life rule. The semi-automated workflow showed higher environmental impacts per test, suggesting that increasing technological complexity does not necessarily translate into proportional environmental benefits. When scaled using the theoretical smoothing rule, the manual workflow had the lowest impact on human health. However, this impact remains quantitatively higher than that of the fully-automated workflow under real-life conditions. These findings indicate that the environmental benefits of automation depend on a scale-dependent threshold, which, based on observed activity levels, likely lies between approximately 5,000 and 20,000 ECUA per year, although this estimate should be interpreted cautiously given the limited number of settings analyzed.

Polystyrene (e.g. agar plates) and polycarbonate (e.g. NMR1 antibiogram plates) materials were major contributors to the environmental footprint of ECUA workflows due to their weight and disposal in infectious waste ("DASRI") streams. The use of multi-layer aluminium packaging for consumables also contributed significantly to the environmental burden. Automated systems were also major contributors, mainly due to energy consumption and electronic components. The fully-automated workflow also included significant impacts from bottled rinse water. These findings are consistent with a study of a Dutch microbiology laboratory identifying consumables, waste processing, and commuting as major emission sources [24].

### 4.2 Recommendations

Optimizing ECUA impact requires an eco-design approach: filtered water systems, lighter recyclable consumables, mono-material options, and minimizing packaging via bulk purchasing or on-site reagent recomposition. Automation energy use should also be optimized by maximizing instrument loads and transitioning to renewable sources of energy.

In addition, waste management strategies should prioritize reducing the use of infectious waste streams when contamination risks are not significant, thereby promoting recycling over incineration or diversion to landfill. Furthermore, hybrid workflows, combining manual cytology with fully-automated standard bacteriology and AST, could optimize performance by leveraging the strengths of each process.

Organizational changes also play a critical role. AST processes represent a major improvement area. Implementing diagnostic stewardship to avoid UA for uncomplicated UTI or initially using dipsticks when pre-test probabilities and likelihood ratios do not justify UA [25], could reduce unnecessary testing and its associated environmental costs. Aligning testing practices with clinical relevance is key, as ∼25% of UA are irrelevant [26,27]. These measures would not only lower emissions but also position healthcare providers as central actors in the environmental transition.

### 4.3 Implications for practice and policy

One of the key contributions of this study is to highlight the role of healthcare professionals in the eco-design of healthcare, thereby strengthening their agency. Many decarbonization levers depend on institutional and policy decisions and are often perceived as outside the scope of frontline professionals, limiting their capacity to act [28]. However, the results show that several levers related to organizational innovation remain actionable at the laboratory level, shaped by daily professional choices. Improving the sustainability of diagnostic testing therefore requires coordinated action involving healthcare professionals alongside hospital managers, purchasing departments, manufacturers, learned societies, and policymakers, rather than individual behavioral change alone. Taken together, these findings highlight that responsibility for implementing mitigation strategies is distributed across multiple levels of the healthcare system. The limited adoption of such strategies appears less related to a lack of awareness than to organizational barriers, particularly insufficient coordination between the levers of action to different actors [29].

Although the values reported here are specific to ECUA, the approach and conclusions can be applied to other routine diagnostic tests with similar characteristics. From this perspective, the present study provides a transferable analytical framework rather than directly generalizable metrics, identifying mechanisms that can inform eco-design efforts across diagnostic activities.

Social factors must be given greater attention in the environmental transition process [30]. Staff transport emerged as a significant emission source, particularly in the manual workflow. Survey data revealed that technical staff often commute longer distances using private vehicles, possibly because of salary constraints. Promoting sustainable commuting options, enhancing public transportation access, and reconsidering laboratory location could mitigate emissions [31].

### 4.4 Limitations and future perspectives

Despite its contributions, the present study has limitations. The analysis excluded the pre-analytical phase, which has been reported to significantly influence the carbon footprint in other contexts, notably in Australia [32], using a different energy mix from that of France [33]. Modeling biomedical instruments using available LCA databases was limited by data availability [34]. Additionally, the lack of data on fixed assets, building infrastructure, and energy use for heating, cooling, servers, or software, especially for AI-driven WASPLab® instruments [35], underestimates the total environmental burden.

Furthermore, this study was designed to identify environmental hotspots and structural levers, but not to quantitatively assess the environmental benefits of specific mitigation or eco-design strategies, which would require dedicated comparative LCA.

While the hybrid LCA approach provided a robust framework, reliance on assumptions and characterization factors introduces inherent uncertainties. These limitations highlight the need for future studies to adopt a full-process perspective integrating more comprehensive data.

Finally, this study underscores the dual carbon constraint facing healthcare systems. This dual challenge necessitates systemic reorganization of healthcare practices to ensure sustainability. This study also highlights the link between workflow material complexity and automation: manual workflows required 18 items, semi-automated 20, and fully-automated 31. Promoting low-tech [36], resource-efficient solutions, developing indicators of medical practice vulnerability, and integrating environmental considerations into clinical guidelines are essential steps toward aligning healthcare systems with environmental imperatives.

## 5 Conclusions

Centralization and automation offer better carbon and DALY performance, showing that ecology, economy, and technology are not antinomic but could synergize. Reducing urine cultures, minimizing antibiograms, and exploring alternative low-impact methods are crucial strategies. These findings emphasize the importance to integrate environmental impact assessments in healthcare to raise awareness and adapt practices to current environmental challenges affecting human health.

## Declaration of generative AI and AI-assisted technologies in the writing process

During the preparation of this work the author used ChatGPT 4o in order to improve language. After using this tool/service, the author reviewed and edited the content as needed and takes full responsibility for the content of the publication.

## Transparency declaration

Conflict of interest:

Julien BRUNIER and Celine RAMANANTSOA have no conflict of interest.

Clara MOURGUES and Fanny AUGER worked at Primum Non Nocere Agency which received funds from Association Nationale pour la Formation permanente du personnel Hospitalier [ANFH] (French National Association for In-Service Training of Hospital Staff) to conduct this study.

François VANDENESCH reports equity and stock ownership in Weezion.

François VANDENESCH, Gérard LINA and Olivier DAUWALDER receive research grants for research projects on automation in microbiology laboratories from bioMérieux.

Olivier DAUWALDER received consulting fees from BD, via the Société Française de Microbiologie (SFM).

Olivier DAUWALDER received non-financial support in the form of reagent gifts for research studies from Hologic, Sysmex, BD, bioMérieux, Coris BioConcept, Thermo Fisher Scientific, NG Biotech and Elitech.

Olivier DAUWALDER received travel grants from Eumedica, Pfizer, bioMérieux, MSD and Advanz for participation in scientific meetings (including ECCMID, RICAI and SFM meetings).

## Funding

This study was supported by Association Nationale pour la Formation permanente du personnel Hospitalier [ANFH] (French National Association for In-Service Training of Hospital Staff).

## CRediT authorship contribution statement

**Julien Brunier:** Writing – review & editing, Writing – original draft, Project administration, Investigation, Funding acquisition, Formal analysis, Data curation, Conceptualization. **Clara Mourgues:** Writing – review & editing, Formal analysis, Data curation. **Fanny Auger:** Writing – review & editing, Methodology, Funding acquisition, Formal analysis, Data curation. **Gérard Lina:** Writing – review & editing. **François Vandenesch:** Writing – review & editing. **Céline Ramanantsoa:** Writing – review & editing, Validation, Supervision, Investigation, Funding acquisition, Data curation. **Olivier Dauwalder:** Writing – review & editing, Writing – original draft, Validation, Supervision, Conceptualization.

## Declaration of competing interest

The authors declare the following financial interests/personal relationships which may be considered as potential competing interests:

Fanny AUGER reports financial support was provided by French National Association for In-Service Training of Hospital Staff.

Olivier Dauwalder reports a relationship with bioMérieux SA that includes: funding grants, non-financial support, and travel reimbursement.

Olivier Dauwalder reports a relationship with Becton Dickinson and Company that includes: consulting or advisory and non-financial support.

Olivier Dauwalder reports a relationship with Hologic Inc that includes: non-financial support.

Olivier Dauwalder reports a relationship with Sysmex Corporation that includes: non-financial support.

Olivier Dauwalder reports a relationship with Coris BioConcept Sprl that includes: non-financial support.

Olivier Dauwalder reports a relationship with Thermo Fisher Scientific Inc that includes: non-financial support.

Olivier Dauwalder reports a relationship with NG Biotech that includes: non-financial support.

Olivier Dauwalder reports a relationship with ELITech Group SAS that includes: non-financial support.

Olivier Dauwalder reports a relationship with Eumedica SA that includes: travel reimbursement.

Olivier Dauwalder reports a relationship with Pfizer Inc that includes: travel reimbursement.

Olivier Dauwalder reports a relationship with MSD France SAS that includes: travel reimbursement.

Olivier Dauwalder reports a relationship with ADVANZ PHARMA Corp. that includes: travel reimbursement.

Fanny Auger reports a relationship with Primum Non Nocere that includes: employment.

Francois Vandenesch reports a relationship with bioMérieux SA that includes: funding grants.

Francois Vandenesch reports a relationship with Weezion that includes: equity or stocks.

Gerard Lina reports a relationship with bioMérieux SA that includes: funding grants.

If there are other authors, they declare that they have no known competing financial interests or personal relationships that could have appeared to influence the work reported in this paper.

## Footnotes

Supplementary material associated with this article can be found, in the online version, at doi:10.1016/j.joclim.2026.100737.
