# Supplementary material for: Environmental impact of urine analyses: Comparison of manual, semi and fully automated analytical workflows
Source: J Clim Chang Health. 2026 Sep 10;31:100737. doi: 10.1016/j.joclim.2026.100737 (PMC13583710; doi:10.1016/j.joclim.2026.100737)
Supplement: Supplementary file 1 [file mmc1.docx]

**Supplementary Table 1.** Life cycle inventory for manual workflow

| **Manual workflow** | |
| --- | --- |
| **Step** | **Equipment** |
| **Recording** | Barcode reader |
|  | Computer |
|  | Work labels |
|  | Work sheet |
| **Cytology** | Microscope |
|  | KOVA glasstic counting slide 10 with grids (Kova International, Garden Grove, United-States of America) |
|  | 10-100μL pipettes |
|  | FlexiBulk tips 200μL (Sartorius, Göttingen, Germany) |
|  | 10μL rigid plastic loop (Copan, Brescia, Italy) |
|  | Closed pasteur pipette, pre-breakable, cotton, 2mL, 230mm (VWR, Radnor, United-States of America) |
|  | Fin plug (Labelians, Nemours, France) |
|  | Latex gloves with hand sanitizer |
| **Streaking** | 10μL rigid plastic loop (Copan, Brescia, Italy) |
|  | Closed pasteur pipette, pre-breakable, cotton, 2mL, 230mm (VWR, Radnor, United-States of America) |
|  | CPSE chromogenic agar plate (bioMérieux, Marcy L’Etoile, France) |
|  | Latex gloves with hand sanitizer |
| **Incubation** | Automation: conventional incubator |
| **Preparation of solid antibiograms** | 0.7 mL saline infusion for McFarland (SIRscan® tubes; I2A, Montpellier, France) |
|  | Inoculation needles (I2A, Montpellier, France) |
|  | Vortexer |
|  | Dryswab in viscose (Copan, Brescia, Italy) |
|  | 2 Mueller-Hinton square agar plate (Bio-Rad, Hercules, United-States of America) |
|  | Labels for agar plate box |
|  | Closed pasteur pipette, pre-breakable, cotton, 2mL, 230mm (VWR, Radnor, United-States of America) |
|  | Columbia agar round plate (Oxoid, Basingstoke, United Kingdom) |
|  | Disc distributor (Bio-Rad, Hercules, United-States of America) |
|  | 32 Antibiotic disc (Bio-Rad, Hercules, United-States of America) |
|  | Hand sanitizer |
| **Incubation** | Automation: conventional incubator |
| **Reading** | Manual reading with calliper |
| **ATB technical interpretation** | CA-SFM/EUCAST |

*Details about reagents, consumables, instruments needed to realize manual workflow in Le Mans laboratory. CPSE = CPS® Elite; CA-SFM = Comité de l’Antibiogramme - Société Française de Microbiologie; EUCAST =* *European Committee on Antimicrobial Susceptibility Testing.*

**Supplementary Table 2.** Life cycle inventory for semi-automated workflow

| **Semi-automated workflow** | |
| --- | --- |
| **Step** | **Equipment** |
| **Recording** | Barcode reader |
|  | Computer |
|  | Work labels |
|  | Work sheet |
| **Cytology** | Automation: Urine sediment analyser (UriSed, I2A, Montpellier, France) |
|  | Counting cells (UriSed cuvettes, 77 Elektronika Kft., Budapest, Hungary) |
|  | 2L demineralized water per day for rinsing (Oxena, Romans-sur-Isère, France) |
|  | 100mL bleach per day for rinsing (McBride SAS, Moyaux, France) |
|  | Fin plug (Labelians, Nemours, France) |
|  | Latex gloves with hand sanitizer |
| **Streaking** | Automation: Automated plates streaker (Prelud, I2A, Montpellier, France) |
|  | CPSE chromogenic agar plate (bioMérieux, Marcy L’Etoile, France) |
|  | Work labels |
|  | Work sheet |
| **Incubation** | Automaton: conventional incubator |
| **Preparation of liquid antibiograms** | Automation: Automated antibiogram plate preparator (SH2000, Beckman Coulter, Brea, United-States of America) |
|  | Prompt inoculation System-D (Beckman Coulter, Brea, United-States of America) |
|  | SH2000 cones (4000μL) (Beckman Coulter, Brea, United-States of America) |
|  | SH2000 waste bag (Beckman Coulter, Brea, United-States of America) |
|  | Neg Multidrug Resistant Minimum Inhibitory Concentration (MIC) 1 (NMR1) plate (Beckman Coulter, Brea, United-States of America) |
|  | WalkAway MicroScan 96Well Tray Lids for cover (Beckman Coulter, Brea, United-States of America) |
|  | Plate labels with printer |
|  | CLED agar round plate (bioMérieux, Marcy L’Etoile, France) |
|  | Dryswab in viscose (Copan, Brescia, Italy) |
|  | Hand sanitizer |
| **Reading** | Automation: automated antibiogram incubator (MicroScan Walkaway 96+, Beckman Coulter, Brea, United-States of America) |
| **ATB technical interpretation** | Automation: antibiogram analyser (SIRscan® 2000 Automatic, I2A, Montpellier, France)  SIRweb Software (I2A, Montpellier, France) |
|  | CA-SFM/EUCAST |

*Details about reagents, consumables, instruments needed to realize semi-automated workflow in Le Mans laboratory.CLED = Cystine Lactose Electrolyt Deficient; CPSE = CPS® Elite; CA-SFM = Comité de l’Antibiogramme - Société Française de Microbiologie; EUCAST =* *European Committee on Antimicrobial Susceptibility Testing*

**Supplementary Table 3.** Life cycle inventory for fully-automated workflow

| **Fully-automated workflow** | |
| --- | --- |
| **Step** | **Equipment** |
| **Recording** | Barcode reader |
|  | Computer |
|  | Work labels |
|  | Work sheet |
| **Cytology** | Automaton: automated urine particle analyser (UF4000®, Sysmex, Kobe, Japan) |
|  | UF-Cellsheath (Sysmex, Kobe, Japan) |
|  | UF-Cellpack SF (Sysmex, Kobe, Japan) |
|  | UF-Cellpack CR (Sysmex, Kobe, Japan) |
|  | UF-Fluorocell SF (Sysmex, Kobe, Japan) |
|  | UF-Fluorocell CR (Sysmex, Kobe, Japan) |
|  | Rinsing water (Aguettant, Lyon, France) |
|  | Hand sanitizer |
| **Streaking** | Automation: automated plate streaker (WASP®, Copan, Brescia, Italy) |
|  | Metallic loop (Copan, Brescia, Italy) |
|  | CPSE chromogenic agar plates (bioMérieux, Marcy L’Etoile, France) |
|  | Plate label |
|  | Loop cleaning liquid (Copan, Brescia, Italy) |
|  | Small sponge (Copan, Brescia, Italy) |
|  | Medium sponge (Copan, Brescia, Italy) |
|  | Large sponge (Copan, Brescia, Italy) |
|  | 2 Trapezoidal sponges (Copan, Brescia, Italy) |
|  | Automation: WASPLab® Conveyor (Copan, Brescia, Italy) |
| **Incubation** | Automation: automated incubator (WASPLab®, Copan, Brescia, Italy) |
| **Preparation of liquid antibiograms** | Automation: automated antibiogram system (Vitek2® XL, bioMérieux, Marcy L’Etoile, France) |
|  | 2 Polystyrene tubes of 5mL (Germany) |
|  | Bottle-top dispensers |
|  | 500mL Distilled water refill (bioMérieux, Marcy L’Etoile, France) |
|  | Plastic pipette 3mL 150mm (Mexico) |
|  | 3 antibiogram labels |
|  | Vitek® 2 AST card N372 (bioMérieux, Marcy L’Etoile, France) |
|  | Columbia Agar round plate (bioMérieux, Marcy L’Etoile, France) |
|  | 10μL plastic loop (Biologix, Changzhou, China) |
|  | Vitek® distilled water pouch refill (bioMérieux, Marcy L’Etoile, France) |
|  | Vitek® straw and tube refill (bioMérieux, Marcy L’Etoile, France) |
|  | Hand sanitizer |
| **Reading** | Automation: WASPLab® automatic incubator with PhenoMatrix Tag® (Copan, Brescia, Italy)  Automation: antibiogram analyser (SIRscan® 2000 Automatic, I2A, Montpellier, France) |
| **ATB technical interpretation** | MYLA® software (bioMérieux, Marcy L’Etoile, France) |
|  | SIRweb Software (I2A, Montpellier, France) |
|  | CA-SFM/EUCAST |

*Details about reagents, consumables, instruments needed to realize fully-automated workflow in Lyon laboratory. CPSE = CPS® Elite; CA-SFM = Comité de l’Antibiogramme - Société Française de Microbiologie; EUCAST = European Committee on Antimicrobial Susceptibility Testing.*

**Supplementary Table 4.** Unitary quantitative environmental impacts (midpoints) and quantitative damage at three key areas of protection (endpoints) for realization of one *Escherichia coli* urinalysis in the three workflows in theoretical scale smoothing rule, according to ReCiPe2016.

|  | Unit | manual workflow | semi-automated workflow | fully-automated workflow |
| --- | --- | --- | --- | --- |
| Midpoints |  |  |  |  |
| Global warming | kgCO_2_eq | 1.857 | 2.387 | **1.428** |
| Stratospheric ozone depletion | kgCFC11eq | 7.5 x10^-7^ | 10.2 x10^-7^ | **6.9 x10^-7^** |
| Ionising radiation | kBqCo-60eq | **0.303** | 0.416 | 0.857 |
| Ozone formation, Human health | kgNOxeq | 4.0 x10^-3^ | 4.8 x10^-3^ | **3.6 x10^-3^** |
| Fine particulate matter formation | kgPM2.5eq | **1.8 x10^-3^** | 2.9 x10^-3^ | 2.2 x10^-3^ |
| Ozone formation, terrestrial eco. | kgNOxeq | 4.1 x10^-3^ | 4.9 x10^-3^ | **3.7 x10^-3^** |
| Terrestrial acidification | kgSO2eq | **4.7 x10^-3^** | 6.5 x10^-3^ | 4.8 x10^-3^ |
| Freshwater eutrophication | kgPeq | **6.3 x10^-4^** | 10.7 x10^-4^ | 9.9 x10^-4^ |
| Marine eutrophication | kgNeq | **3.7 x10^-5^** | 5.3 x10^-5^ | 8.7 x10^-5^ |
| Terrestrial ecotoxicity | kg1,4-DCB | 6.169 | 6.137 | **6.078** |
| Freshwater ecotoxicity | kg1,4-DCB | **0.102** | 0.303 | 0.352 |
| Marine ecotoxicity | kg1,4-DCB | **0.134** | 0.399 | 0.461 |
| Human carcinogenic toxicity | kg1,4-DCB | 0.137 | 0.174 | **0.123** |
| Human non-carcinogenic toxicity | kg1,4-DCB | **1.606** | 4.784 | 5.308 |
| Land use | m^2^acropeq | 0.084 | 0.082 | **0.080** |
| Mineral resource scarcity | kgCueq | **0.005** | 0.017 | 0.018 |
| Fossil resource scarcity | kgoileq | 0.521 | 0.610 | **0.433** |
| Water consumption | m^3^ | **0.014** | 0.017 | 0.016 |
| Endpoints | | | | |
| Human health | DALY | **3.7 x10^-6^** | 5.8 x10^-6^ | 4.4 x10^-6^ |
| Ecosystems | species.yr | 8.2 x10^-9^ | 10.6 x10^-9^ | **7.3 x10^-9^** |
| Resources | USD2013 | 0.203 | 0.207 | **0.144** |

*Units are expressed in terms of kilograms (kg) per equivalent (eq) of carbon dioxide to air (CO_2_), of trichlorofluoromethane to air (CFC11), of cobalt-60 to air (Co-60), of nitrogen oxides to air (NOx), of particulate matter of 2.5 micrometres or smaller to air (PM2.5), of sulphur dioxide to air (SO_2_), of potassium to freshwater (P), of nitrogen to marine water (N) of 1,4-dichlorobenzene to industrial soil, to freshwater, to marine water, to urban air (1,4-DCB); in terms of square meters of annual cropland (m^2^acrop); in terms of kilograms per equivalent of copper (Cu), of oil; in terms of cubic meters of water (m^3^), in terms of disability-adjusted life years (DALY); in terms of number of species lost (species.yr); in terms of surplus costs of future resource production (USD2013). The lowest impact between the three workflows is indicated in bold.*

**Supplementary Table 5.** Synthesis of mobility survey.

| ***Global overview*** | | | |
| --- | --- | --- | --- |
| Transportation mode | People, n | Round-trip distance km | Proportion of total distance, % |
| Single car | 23 | 394.7 | 57.5 |
| Motorbike | 1 | 10.0 | 1.5 |
| Carpooling | 4 | 88.5 | 12.9 |
| Urban public transport | 6 | 84.5 | 12.3 |
| Train | 1 | 100.0 | 14.6 |
| Conventional bicycle | 2 | 6.0 | 0.9 |
| Walking | 2 | 2.5 | 0.4 |
| Total | 39 | 686.2 | 100.0 |
| ***Overview for laboratory technicians only*** | | | |
| Transportation mode | People, n | Round-trip distance km | Proportion of total distance, % |
| Single car | 20 | 363.7 | 66.6 |
| Motorbike | 1 | 10.0 | 1.8 |
| Carpooling | 4 | 88.5 | 16.2 |
| Urban public transport | 4 | 79.5 | 14.6 |
| Train | 0 | 0 | 0.00 |
| Conventional bicycle | 1 | 3.0 | 0.6 |
| Walking | 1 | 1.5 | 0.3 |
| Total | 31 | 546.2 | 100.0 |

The number of people using different modes of transport (single car, motorbike, carpooling, urban public transport [bus, metro, tram], train, conventional bicycle, walking) is combined with the total mileage for each mode. The proportion of the total distance covered by the team for each mode of transport is also calculated, enabling a modal split to be achieved.

**Supplementary Figure 1:** Details of the impact categories covered by the ReCiPe2016 method and their relationship with damage pathways and area of protection, adapted from *ReCiPe2016: a harmonized life cycle impact assessment method at midpoint and endpoint level* [18]*.*

*Each environmental impact category can influence the areas of protection through different mechanisms and therefore several pathways. Details of these influences can be found in the source. The dotted line indicates that there is no constant mid-to-endpoint factor for fossil resources.*

Relative impact on workflow (%)

|  | Polystyrene | Water, ultrapure | Polyethylene, low density, granulate | Solid bleached and unbleached board carton | Transport, freight, sea, container ship | Transport, freight, lorry 16-32 metric ton | Transport, freight, lorry 16-32 metric ton | Hazardous waste, for incineration | Municipal solid waste, incineration |
| --- | --- | --- | --- | --- | --- | --- | --- | --- | --- |
| Global warming | 33.1235 | 0.0351446 | 0.327557 | 0.920439 | 3.34774 | 10.6998 | 0.0258268 | 50.6763 | 0.843785 |
| Stratospheric ozone depletion | 1.74124 | 0.132072 | 0.188419 | 2.48229 | 8.03599 | 27.7555 | 0.0669956 | 56.5971 | 3.00036 |
| Ionizing radiation | 1.07575 | 0.954062 | 0.750045 | 29.0954 | 2.05638 | 15.1312 | 0.0365232 | 50.8208 | 0.0798944 |
| Ozone formation, Human health | 27.9826 | 0.0305789 | 0.371206 | 1.87388 | 33.3755 | 16.6623 | 0.040219 | 19.4166 | 0.247229 |
| Fine particulate matter formation | 32.1563 | 0.0576906 | 0.457079 | 2.06586 | 25.255 | 13.1179 | 0.0316637 | 26.7446 | 0.113918 |
| Ozone formation, Terrestrial ecosystems | 28.836 | 0.0302238 | 0.397116 | 1.87066 | 32.7165 | 16.6543 | 0.0401997 | 19.2132 | 0.241835 |
| Terrestrial acidification | 36.164 | 0.0506825 | 0.381991 | 1.7949 | 28.5958 | 10.9495 | 0.0264297 | 21.925 | 0.111686 |
| Freshwater eutrophication | 8.1677 | 1.98631 | 0.296418 | 3.09539 | 1.67089 | 7.26073 | 0.0175258 | 76.4204 | 1.0846 |
| Marine eutrophication | 14.7653 | 6.1906 | 0.555377 | 6.2198 | 0.942406 | 5.10456 | 0.0123213 | 64.7846 | 1.42507 |
| Terrestrial ecotoxicity | 7.00482 | 0.0226774 | 0.224378 | 1.90026 | 3.09434 | 67.3463 | 0.162559 | 20.1035 | 0.141171 |
| Freshwater ecotoxicity | 9.44521 | 0.0872332 | 0.437061 | 2.39133 | 1.56667 | 11.7766 | 0.0284262 | 49.5804 | 24.6871 |
| Marine ecotoxicity | 9.37055 | 0.0832265 | 0.412406 | 2.2923 | 1.67946 | 15.1931 | 0.0366727 | 48.1289 | 22.8034 |
| Human carcinogenic toxicity | 8.19651 | 0.103095 | 0.173745 | 1.05432 | 2.14544 | 6.24148 | 0.0150655 | 81.3002 | 0.770197 |
| Human non-carcinogenic toxicity | 9.41118 | 0.101759 | 0.43277 | 2.46367 | 1.12783 | 17.5812 | 0.0424372 | 50.3417 | 18.4974 |
| Land use | 1.32815 | 0.0239993 | 0.0927505 | 71.9936 | 1.2541 | 15.1125 | 0.0364781 | 10.1338 | 0.024712 |
| Mineral resource scarcity | 7.98478 | 0.160394 | 0.659988 | 8.88756 | 8.35111 | 25.6552 | 0.061926 | 47.8247 | 0.414321 |
| Fossil resource scarcity | 60.012 | 0.0330596 | 0.827246 | 1.11871 | 3.725 | 13.8906 | 0.0335287 | 20.3136 | 0.0462801 |
| Water consumption | 64.9454 | 2.082 | 0.606862 | 2.9563 | 0.367057 | 2.65789 | 0.00641555 | 26.101 | 0.277108 |

**Supplementary Figure 2:** Life cycle assessment of Mueller-Hinton square agar plate (Bio-Rad, Hercules, United-States of America).

Relative impact on workflow (%)

|  | Polycarbonate | Aluminium alloy, metal matrix composite | Activated silica | Solid bleached and unbleached board carton | Transport, freight, lorry 16-32 metric ton | Transport, freight, sea, container ship | Transport, freight, lorry 16-32 metric ton | Hazardous waste, for incineration | Municipal solid waste, incineration |
| --- | --- | --- | --- | --- | --- | --- | --- | --- | --- |
| Global warming | 60.6071 | 15.9618 | 0.159531 | 0.787006 | 3.52807 | 0.521938 | 0.0107376 | 17.2667 | 1.15713 |
| Stratospheric ozone depletion | 59.415 | 12.0182 | 0.140716 | 1.67807 | 7.23579 | 0.990558 | 0.022022 | 15.2466 | 3.25309 |
| Ionizing radiation | 1.17347 | 43.8984 | 0.466942 | 28.4467 | 5.70504 | 0.366601 | 0.0173632 | 19.8002 | 0.125282 |
| Ozone formation, Human health | 53.6536 | 23.2824 | 0.256479 | 1.89623 | 6.50225 | 6.1583 | 0.0197894 | 7.82967 | 0.401249 |
| Fine particulate matter formation | 52.7082 | 31.1062 | 0.461791 | 1.43824 | 3.52189 | 3.20599 | 0.0107188 | 7.41973 | 0.1272 |
| Ozone formation, Terrestrial ecosystems | 54.2223 | 22.8852 | 0.254038 | 1.89715 | 6.51344 | 6.05002 | 0.0198235 | 7.76474 | 0.393359 |
| Terrestrial acidification | 55.6892 | 27.6308 | 0.558148 | 1.43533 | 3.37667 | 4.16965 | 0.0102768 | 6.98674 | 0.143244 |
| Freshwater eutrophication | 16.5861 | 34.5737 | 0.440573 | 3.90133 | 3.52905 | 0.383997 | 0.0107406 | 38.3821 | 2.19247 |
| Marine eutrophication | 7.60651 | 45.7404 | 0.434691 | 7.88277 | 2.49483 | 0.217783 | 0.00759295 | 32.7187 | 2.8967 |
| Terrestrial ecotoxicity | 13.7652 | 23.2701 | 2.32195 | 3.1355 | 42.8536 | 0.93099 | 0.130424 | 13.2186 | 0.373597 |
| Freshwater ecotoxicity | 9.89757 | 33.1012 | 1.72346 | 1.98526 | 3.77033 | 0.237159 | 0.0114749 | 16.4025 | 32.871 |
| Marine ecotoxicity | 10.1779 | 32.9729 | 1.6586 | 1.96976 | 5.03464 | 0.263146 | 0.0153228 | 16.4805 | 31.4273 |
| Human carcinogenic toxicity | 20.5429 | 42.7653 | 0.240517 | 1.02503 | 2.34009 | 0.380335 | 0.00712202 | 31.4977 | 1.20097 |
| Human non-carcinogenic toxicity | 10.3369 | 38.4819 | 1.43505 | 2.07031 | 5.69747 | 0.172815 | 0.0173401 | 16.8579 | 24.9304 |
| Land use | 1.46118 | 8.63707 | 0.309876 | 78.5194 | 6.35621 | 0.249401 | 0.019345 | 4.40428 | 0.0432272 |
| Mineral resource scarcity | 2.31277 | 82.66 | 1.06385 | 3.09814 | 3.44885 | 0.53082 | 0.0104965 | 6.64344 | 0.231644 |
| Fossil resource scarcity | 69.0279 | 15.3876 | 0.167285 | 1.12434 | 5.3837 | 0.682639 | 0.0163852 | 8.13559 | 0.0746003 |
| Water consumption | 64.4894 | 19.9276 | 0.517552 | 2.98978 | 1.03648 | 0.0676949 | 0.00315449 | 10.5189 | 0.449427 |

**Supplementary Figure 3:** Life cycle assessment of Neg Multidrug Resistant Minimum Inhibitory Concentration (MIC) 1 (NMR1) plate (Beckman Coulter, Brea, United-States of America)for WalkAway instrument.

Relative impact on workflow (%)

|  | Steel, low-alloyed | Polyethylene, high density, granulate | Electronic component, active, unspecified | Electricity, low voltage | Used industrial electronic device |
| --- | --- | --- | --- | --- | --- |
| Global warming | 2.24674 | 0.513192 | 88.578 | 8.20911 | 0.452947 |
| Stratospheric ozone depletion | 0.883659 | 0.156614 | 82.1652 | 16.269 | 0.525538 |
| Ionizing radiation | 0.112449 | 0.0196447 | 14.952 | 84.903 | 0.0129903 |
| Ozone formation, Human health | 1.97677 | 0.380459 | 91.1273 | 6.41176 | 0.103709 |
| Fine particulate matter formation | 2.02693 | 0.255681 | 91.7471 | 5.88773 | 0.0825581 |
| Ozone formation, Terrestrial ecosystems | 2.07562 | 0.401071 | 91.0089 | 6.41148 | 0.102936 |
| Terrestrial acidification | 1.6612 | 0.342185 | 89.6613 | 8.25536 | 0.0799157 |
| Freshwater eutrophication | 1.09959 | 0.0896009 | 96.1679 | 2.58478 | 0.0581521 |
| Marine eutrophication | 2.31702 | 0.122676 | 79.0592 | 18.3726 | 0.128485 |
| Terrestrial ecotoxicity | 3.38277 | 0.263031 | 71.4688 | 24.5274 | 0.358026 |
| Freshwater ecotoxicity | 0.382285 | 0.0221808 | 93.0366 | 5.86585 | 0.693053 |
| Marine ecotoxicity | 0.40741 | 0.0224977 | 93.4295 | 5.4842 | 0.656423 |
| Human carcinogenic toxicity | 32.178 | 0.214662 | 58.5319 | 8.9262 | 0.149262 |
| Human non-carcinogenic toxicity | 0.317286 | 0.0320344 | 96.8511 | 2.67713 | 0.122415 |
| Land use | 1.5985 | 0.152846 | 87.321 | 10.8509 | 0.0767532 |
| Mineral resource scarcity | 4.35918 | 0.0323066 | 91.9049 | 3.69316 | 0.0104869 |
| Fossil resource scarcity | 2.02937 | 1.41243 | 87.8283 | 8.6501 | 0.079813 |
| Water consumption | 1.61491 | 0.430426 | 70.1743 | 27.7186 | 0.0618067 |

**Supplementary Figure 4:** Life cycle assessment of automated antibiogram incubator (MicroScan Walkaway 96+, Beckman Coulter, Brea, United-States of America)

Relative impact on workflow (%)

|  | Steel, low-alloyed | Polyethylene, high density, granulate | Electronic component, active, unspecified | Electricity, low voltage | Used industrial electronic device |
| --- | --- | --- | --- | --- | --- |
| Global warming | 0.852824 | 0.194804 | 33.6231 | 65.1573 | 0.17193 |
| Stratospheric ozone depletion | 0.208449 | 0.036945 | 19.3824 | 80.2482 | 0.12397 |
| Ionizing radiation | 0.00628054 | 0.00109723 | 0.835108 | 99.1568 | 0.000725531 |
| Ozone formation, Human health | 0.868296 | 0.167121 | 40.0281 | 58.8909 | 0.0455538 |
| Fine particulate matter formation | 0.933092 | 0.117705 | 42.236 | 56.6752 | 0.0380051 |
| Ozone formation, Terrestrial ecosystems | 0.911737 | 0.176179 | 39.9771 | 58.8898 | 0.0452155 |
| Terrestrial acidification | 0.628368 | 0.129438 | 33.9158 | 65.2962 | 0.0302288 |
| Freshwater eutrophication | 0.725968 | 0.0591578 | 63.4926 | 35.6838 | 0.0383929 |
| Marine eutrophication | 0.497425 | 0.026337 | 16.9728 | 82.4758 | 0.0275834 |
| Terrestrial ecotoxicity | 0.574961 | 0.044708 | 12.1475 | 87.172 | 0.0608525 |
| Freshwater ecotoxicity | 0.176338 | 0.0102316 | 42.9157 | 56.5781 | 0.319684 |
| Marine ecotoxicity | 0.194753 | 0.0107548 | 44.6624 | 54.8183 | 0.313786 |
| Human carcinogenic toxicity | 11.5863 | 0.0772952 | 21.0758 | 67.2068 | 0.0537443 |
| Human non-carcinogenic toxicity | 0.206966 | 0.0208966 | 63.1768 | 36.5155 | 0.0798511 |
| Land use | 0.505781 | 0.0483631 | 27.6295 | 71.7921 | 0.0242853 |
| Mineral resource scarcity | 2.51202 | 0.0186175 | 52.9617 | 44.5017 | 0.00604315 |
| Fossil resource scarcity | 0.745472 | 0.518856 | 32.2633 | 66.4431 | 0.0293184 |
| Water consumption | 0.24773 | 0.0660296 | 10.765 | 88.9118 | 0.00948117 |

**Supplementary Figure 5:** Life cycle assessment of automated antibiogram system (Vitek2® XL, bioMérieux, Marcy L’Etoile, France).

Relative impact on workflow (%)

|  | Water, ultrapure | Polypropylene, granulate | Polyethylene, high density, granulate | Solid bleached and unbleached board carton | Transport, freight, lorry 16-32 metric ton | Transport, freight, lorry 16-32 metric ton | Municipal solid waste, incineration | Wastewater, average |
| --- | --- | --- | --- | --- | --- | --- | --- | --- |
| Global warming | 1.00947 | 46.5439 | 4.49142 | 3.53641 | 37.4045 | 0.0317012 | 6.81063 | 0.172016 |
| Stratospheric ozone depletion | 2.37013 | 13.1367 | 1.66131 | 5.95867 | 60.6217 | 0.0513784 | 15.1307 | 1.06944 |
| Ionizing radiation | 10.7215 | 21.5606 | 2.55498 | 43.7358 | 20.6951 | 0.0175396 | 0.2523 | 0.462209 |
| Ozone formation, Human health | 0.737908 | 38.4191 | 3.88341 | 6.04865 | 48.9364 | 0.0414749 | 1.6765 | 0.25659 |
| Fine particulate matter formation | 1.41353 | 46.7207 | 4.70571 | 6.77074 | 39.1185 | 0.033154 | 0.784359 | 0.453328 |
| Ozone formation, Terrestrial ecosystems | 0.718972 | 39.214 | 3.98891 | 5.95243 | 48.2176 | 0.0408657 | 1.61661 | 0.250553 |
| Terrestrial acidification | 1.3844 | 49.4584 | 4.80446 | 6.5581 | 36.4012 | 0.0308509 | 0.857283 | 0.505384 |
| Freshwater eutrophication | 39.8708 | 23.6568 | 2.33217 | 8.3111 | 17.738 | 0.0150334 | 6.11789 | 1.95811 |
| Marine eutrophication | 39.2984 | 11.8876 | 1.14811 | 5.28146 | 3.94382 | 0.00334249 | 2.54215 | 35.8951 |
| Terrestrial ecotoxicity | 0.231163 | 11.8113 | 1.20346 | 2.59104 | 83.5518 | 0.0708123 | 0.404383 | 0.136031 |
| Freshwater ecotoxicity | 0.788692 | 18.5342 | 1.80644 | 2.89201 | 12.9588 | 0.0109829 | 62.7217 | 0.287248 |
| Marine ecotoxicity | 0.770143 | 17.942 | 1.74935 | 2.83737 | 17.1109 | 0.0145019 | 59.2969 | 0.2789 |
| Human carcinogenic toxicity | 4.69233 | 38.549 | 4.36339 | 6.41883 | 34.5744 | 0.0293027 | 9.85086 | 1.52192 |
| Human non-carcinogenic toxicity | 1.02103 | 19.2602 | 1.89118 | 3.30663 | 21.4701 | 0.0181965 | 52.1558 | 0.876807 |
| Land use | 0.200969 | 3.26848 | 0.336614 | 80.6416 | 15.4022 | 0.0130537 | 0.0581515 | 0.0789831 |
| Mineral resource scarcity | 1.80833 | 43.0296 | 3.64403 | 13.4031 | 35.2031 | 0.0298356 | 1.31265 | 1.56935 |
| Fossil resource scarcity | 0.490941 | 65.737 | 6.15855 | 2.2222 | 25.1055 | 0.0212775 | 0.19313 | 0.0714509 |
| Water consumption | 40.033 | 40.2167 | 4.42411 | 7.60363 | 6.22 | 0.00527161 | 1.4973 | -30.6988 |

**Supplementary Figure 6:** Life cycle assessment of rinsing water automated urine particle analyser (UF4000®, Sysmex, Kobe, Japan)

Relative impact on workflow (%)

|  | Steel, low-alloyed | Polyethylene, high density, granulate | Electronic component, active, unspecified | Electricity, low voltage | Used industrial electronic device |
| --- | --- | --- | --- | --- | --- |
| Global warming | 1.32787 | 0.303332 | 52.3453 | 45.7558 | 0.267696 |
| Stratospheric ozone depletion | 0.372616 | 0.0660451 | 34.6428 | 64.6969 | 0.221601 |
| Ionizing radiation | 0.0137841 | 0.00240824 | 1.83259 | 98.1496 | 0.00159232 |
| Ozone formation, Human health | 1.28324 | 0.246998 | 59.1492 | 39.2532 | 0.0673223 |
| Fine particulate matter formation | 1.35466 | 0.170892 | 61.3099 | 37.1094 | 0.0551747 |
| Ozone formation, Terrestrial ecosystems | 1.34743 | 0.260384 | 59.0733 | 39.2521 | 0.0668217 |
| Terrestrial acidification | 0.979549 | 0.20179 | 52.8638 | 45.9078 | 0.0471224 |
| Freshwater eutrophication | 0.902927 | 0.0735817 | 78.959 | 20.0167 | 0.0477507 |
| Marine eutrophication | 0.909047 | 0.0481336 | 31.0139 | 67.9785 | 0.0504081 |
| Terrestrial ecotoxicity | 1.10269 | 0.0857475 | 23.294 | 75.4008 | 0.116704 |
| Freshwater ecotoxicity | 0.255808 | 0.0148435 | 62.2484 | 37.0172 | 0.463749 |
| Marine ecotoxicity | 0.278619 | 0.0153869 | 63.8868 | 35.3703 | 0.448904 |
| Human carcinogenic toxicity | 18.3615 | 0.1225 | 33.3956 | 48.0353 | 0.0851701 |
| Human non-carcinogenic toxicity | 0.258885 | 0.0261401 | 79.0149 | 20.6002 | 0.0998808 |
| Land use | 0.834852 | 0.0798333 | 45.5999 | 53.4453 | 0.0400852 |
| Mineral resource scarcity | 3.32444 | 0.02464 | 70.0811 | 26.5618 | 0.00799747 |
| Fossil resource scarcity | 1.17362 | 0.816892 | 50.7863 | 47.177 | 0.046156 |
| Water consumption | 0.483971 | 0.129004 | 21.0279 | 78.3406 | 0.0185224 |

**Supplementary Figure 7:** Life cycle assessment of conventional incubator

Relative impact on workflow (%)

|  | Microscope | Urinalysis microscope slide | 200µL tips | Fin plug | Plastic loops | Pasteur pipettes | Chromogenic agar plate | Incubator | Saline infusion | Inoculation needles | Dryswab | Square agar plates | Pasteur pipette | Columbia agar plate | Antibiotic disc | Vortexer | Hand sanitiser | Transport |
| --- | --- | --- | --- | --- | --- | --- | --- | --- | --- | --- | --- | --- | --- | --- | --- | --- | --- | --- |
| Global warming | 0.374975 | 3.68274 | 0.223914 | 0.162975 | 0.76129 | 0.636283 | 7.42108 | 4.60948 | 1.67545 | 0.243671 | 1.55305 | 51.9271 | 0.636283 | 8.81192 | 0.856833 | 0.289307 | 0.771862 | 15.3618 |
| Stratospheric ozone depletion | 0.501045 | 2.24542 | 2.2613 | 0.122889 | 0.670902 | 0.904348 | 4.27031 | 9.02352 | 11.9391 | 0.223636 | 0.925207 | 37.3855 | 0.904348 | 6.32356 | 0.938163 | 0.370901 | 2.56131 | 18.4285 |
| Ionizing radiation | 0.853384 | 0.425236 | 0.0374218 | 0.020441 | 0.329293 | 0.120818 | 0.811435 | 86.005 | 0.361725 | 0.0422945 | 0.198294 | 5.29039 | 0.120818 | 0.954542 | 0.304729 | 0.245056 | 0.0574269 | 3.82173 |
| Ozone formation, Human health | 0.509398 | 3.3981 | 0.210467 | 0.163231 | 0.625029 | 0.686248 | 4.43381 | 5.64662 | 1.75708 | 0.31274 | 1.08665 | 50.3297 | 0.686248 | 8.44331 | 1.04588 | 0.39639 | 0.689228 | 19.5798 |
| Fine particulate matter formation | 0.825017 | 3.15397 | 0.241917 | 0.156747 | 0.621804 | 0.763096 | 4.83977 | 8.85826 | 1.83767 | 0.313025 | 1.19883 | 45.7743 | 0.763096 | 7.65465 | 1.09224 | 0.643562 | 0.711966 | 20.5501 |
| Ozone formation, Terrestrial ecosystems | 0.502262 | 3.43046 | 0.213669 | 0.164728 | 0.630529 | 0.675498 | 4.48034 | 5.56742 | 1.75385 | 0.320235 | 1.10261 | 50.2056 | 0.675498 | 8.41288 | 1.04454 | 0.390837 | 0.746923 | 19.6821 |
| Terrestrial acidification | 0.601874 | 3.62437 | 0.230962 | 0.157103 | 0.644789 | 0.795651 | 5.09854 | 7.41764 | 1.74149 | 0.282645 | 1.214 | 49.4195 | 0.795651 | 8.24771 | 0.974276 | 0.464269 | 0.744924 | 17.5446 |
| Freshwater eutrophication | 1.40853 | 1.6626 | 0.178384 | 0.150015 | 0.747208 | 0.737454 | 6.64169 | 12.097 | 1.88319 | 0.238886 | 1.39733 | 44.491 | 0.737454 | 7.45903 | 0.992274 | 1.11532 | 0.82392 | 17.2387 |
| Marine eutrophication | 1.07294 | 1.93071 | 0.251095 | 0.1158 | 0.810385 | 5.15719 | 5.05987 | 21.0159 | 2.04438 | 0.28018 | 1.22341 | 32.4811 | 5.15719 | 5.57125 | 1.06522 | 0.784967 | 0.472075 | 15.5063 |
| Terrestrial ecotoxicity | 0.431776 | 2.52876 | 0.240225 | 0.147615 | 0.622801 | 0.615741 | 3.16735 | 10.5478 | 2.07817 | 0.301301 | 0.941318 | 44.4888 | 0.615741 | 7.48778 | 1.53236 | 0.304441 | 0.651864 | 23.2962 |
| Freshwater ecotoxicity | 3.63425 | 4.71074 | 0.424458 | 0.0610822 | 0.996512 | 0.335402 | 2.67715 | 38.9689 | 1.21107 | 0.341619 | 0.603163 | 16.8125 | 0.335402 | 3.07014 | 0.853024 | 2.83525 | 0.553925 | 21.5755 |
| Marine ecotoxicity | 3.6256 | 4.71094 | 0.421943 | 0.0640762 | 0.998763 | 0.341225 | 2.75429 | 37.9622 | 1.23718 | 0.346422 | 0.62216 | 17.9436 | 0.341225 | 3.25575 | 0.87685 | 2.83351 | 0.567071 | 21.0972 |
| Human carcinogenic toxicity | 0.515303 | 1.39043 | 0.147362 | 0.161504 | 0.577096 | 0.719103 | 7.24784 | 6.58485 | 1.65192 | 0.767143 | 1.29676 | 48.8634 | 0.719103 | 8.09699 | 2.10671 | 0.396071 | 0.373658 | 18.3847 |
| Human non-carcinogenic toxicity | 3.58136 | 5.50196 | 0.497862 | 0.0908906 | 1.20046 | 0.480018 | 3.70993 | 30.9699 | 1.75003 | 0.397947 | 0.86154 | 24.9435 | 0.480018 | 4.46855 | 1.09251 | 2.83469 | 0.618946 | 16.5199 |
| Land use | 0.265865 | 7.45586 | 0.0675112 | 0.0448715 | 6.23864 | 1.38808 | 7.70838 | 3.75169 | 1.88179 | 0.086245 | 1.16143 | 43.2696 | 1.38808 | 8.63581 | 4.74327 | 0.20248 | 0.195513 | 11.5149 |
| Mineral resource scarcity | 3.80947 | 0.905154 | 0.218593 | 0.0788817 | 0.40673 | 0.396628 | 1.87442 | 35.4318 | 1.36988 | 0.69449 | 0.523167 | 15.6861 | 0.396628 | 2.69203 | 2.45329 | 3.00149 | 0.645664 | 29.4156 |
| Fossil resource scarcity | 0.34085 | 5.2098 | 0.377278 | 0.229259 | 0.837328 | 0.40665 | 6.90706 | 4.29221 | 1.88848 | 0.357395 | 1.95152 | 49.0992 | 0.40665 | 8.44862 | 0.982497 | 0.262418 | 1.55541 | 16.4474 |
| Water consumption | 0.526164 | 5.30236 | 0.284369 | 0.132089 | 0.986272 | 1.58236 | 7.46863 | 14.2487 | 1.72236 | 0.267783 | 1.72619 | 46.3941 | 1.58236 | 8.05629 | 0.841894 | 0.363365 | 0.771349 | 7.74334 |

**Supplementary Figure 8.** Relative environmental impacts of manual workflow in real-life at Le Mans laboratory analysed using the 18 indicators of Product Environmental Footprint method and distributed between reagents, consumables, instruments, and human resources used.

Relative impact on workflow (%)

|  | Urine sediment analyser | Counting cells | Demineralised water | Bleach | Fin plug | Automated plates streaker | Chromogenic agar plate | Incubator | Automated antibiogram plate preparator | Inoculation system | SH2000 tips | SH2000 waste bag | NMR1 antibiogram plate | Well tray lids | CLED agar plate | Dryswab | Automated antibiogram incubator | Antibiogram analyser | Hand sanitiser | Transport |
| --- | --- | --- | --- | --- | --- | --- | --- | --- | --- | --- | --- | --- | --- | --- | --- | --- | --- | --- | --- | --- |
| Global warming | 0.964348 | 0.251563 | 0.218329 | 0.0160526 | 0.114605 | 4.5092 | 5.21855 | 1.62071 | 4.76194 | 4.7157 | 0.706828 | 4.62165 | 33.2161 | 8.12121 | 5.16781 | 1.09212 | 12.4662 | 3.75336 | 0.361852 | 8.10187 |
| Stratospheric ozone depletion | 1.17497 | 0.259861 | 0.291547 | 0.0271888 | 0.079867 | 5.3854 | 2.77533 | 2.93226 | 5.63557 | 4.17649 | 5.31929 | 3.03957 | 27.9549 | 6.96159 | 2.69007 | 0.601305 | 16.0918 | 4.51055 | 1.10976 | 8.98269 |
| Ionizing radiation | 1.69405 | 0.0421867 | 0.0292965 | 0.00950643 | 0.0140005 | 4.9031 | 0.555771 | 29.4534 | 3.74229 | 0.814977 | 0.186182 | 0.774026 | 2.88256 | 0.366133 | 0.56438 | 0.135817 | 46.9873 | 4.85557 | 0.0262221 | 1.9632 |
| Ozone formation, Human health | 1.34275 | 0.190739 | 0.277778 | 0.0175298 | 0.117316 | 6.30436 | 3.18665 | 2.02916 | 6.66998 | 3.75391 | 0.77514 | 4.20227 | 27.8027 | 6.07281 | 3.2072 | 0.780994 | 17.1432 | 5.24099 | 0.330239 | 10.5542 |
| Fine particulate matter formation | 1.60823 | 0.172641 | 0.167808 | 0.0175645 | 0.0832435 | 7.55972 | 2.57026 | 2.35218 | 8.00235 | 2.66098 | 0.607473 | 3.2535 | 27.158 | 5.38997 | 2.58079 | 0.636663 | 20.459 | 6.28233 | 0.252069 | 8.18517 |
| Ozone formation, Terrestrial ecosystems | 1.32891 | 0.192646 | 0.281091 | 0.0176572 | 0.118837 | 6.2394 | 3.23219 | 2.00821 | 6.60126 | 3.74972 | 0.778258 | 4.35988 | 27.7772 | 6.10196 | 3.25538 | 0.795437 | 16.9665 | 5.18698 | 0.359228 | 10.6492 |
| Terrestrial acidification | 1.37861 | 0.189505 | 0.185201 | 0.0175555 | 0.0984012 | 6.44557 | 3.19347 | 2.32301 | 6.8065 | 3.02593 | 0.700542 | 3.53784 | 30.1062 | 6.26537 | 3.2206 | 0.760386 | 17.8271 | 5.36532 | 0.311055 | 8.24178 |
| Freshwater eutrophication | 2.32312 | 0.145568 | 0.0933652 | 0.0170221 | 0.0670699 | 10.9988 | 2.96942 | 2.70422 | 11.68 | 3.53789 | 0.472979 | 2.92079 | 12.275 | 2.23874 | 2.88553 | 0.62473 | 28.8995 | 9.12023 | 0.245576 | 5.78039 |
| Marine eutrophication | 2.15795 | 0.22009 | 0.334113 | 0.0468169 | 0.064792 | 9.83483 | 2.83107 | 5.87936 | 10.2645 | 3.27825 | 0.654608 | 3.26565 | 11.1531 | 1.59901 | 2.77642 | 0.684515 | 30.02 | 8.25185 | 0.176089 | 6.50698 |
| Terrestrial ecotoxicity | 1.34893 | 0.293015 | 0.923272 | 0.0341071 | 0.129795 | 6.03863 | 2.785 | 4.63723 | 6.24919 | 5.93719 | 0.784076 | 4.70429 | 18.4392 | 3.613 | 2.74234 | 0.827684 | 19.6727 | 5.0954 | 0.382115 | 15.3629 |
| Freshwater ecotoxicity | 3.09231 | 0.0654481 | 0.0758943 | 0.00823488 | 0.0141589 | 14.5365 | 0.620564 | 4.5165 | 15.3879 | 0.784522 | 0.193244 | 0.605597 | 3.65109 | 0.454325 | 0.604708 | 0.139813 | 39.3326 | 12.08 | 0.0856 | 3.75091 |
| Marine ecotoxicity | 3.09195 | 0.0661673 | 0.0810576 | 0.00826867 | 0.0148779 | 14.5472 | 0.639519 | 4.40722 | 15.4051 | 0.821432 | 0.194738 | 0.630688 | 3.77109 | 0.479973 | 0.62329 | 0.144459 | 39.2256 | 12.0857 | 0.0877789 | 3.67393 |
| Human carcinogenic toxicity | 1.28462 | 0.215648 | 0.126084 | 0.0136186 | 0.110245 | 5.99702 | 4.94747 | 2.24745 | 6.32838 | 5.72711 | 0.686095 | 4.2937 | 26.6838 | 4.39515 | 4.79144 | 0.885187 | 16.6903 | 4.99442 | 0.170043 | 9.41225 |
| Human non-carcinogenic toxicity | 3.06074 | 0.0826797 | 0.10465 | 0.0103688 | 0.0210592 | 14.4882 | 0.859584 | 3.58783 | 15.3842 | 1.11468 | 0.245206 | 0.910795 | 5.34912 | 0.663453 | 0.838308 | 0.199617 | 38.0989 | 12.0143 | 0.0956057 | 2.87071 |
| Land use | 0.9008 | 0.200275 | 0.220937 | 0.0113175 | 0.0417485 | 4.1858 | 7.17189 | 1.74529 | 4.40793 | 10.4155 | 2.40801 | 2.42311 | 28.7095 | 4.87915 | 7.68801 | 1.0806 | 11.8629 | 3.4909 | 0.121271 | 8.0351 |
| Mineral resource scarcity | 2.98588 | 0.04733 | 0.0440798 | 0.00563822 | 0.0167882 | 14.1033 | 0.398927 | 3.77042 | 14.9611 | 0.628223 | 0.188799 | 0.667061 | 7.44865 | 0.314571 | 0.395837 | 0.111344 | 37.4222 | 11.703 | 0.0916099 | 4.69534 |
| Fossil resource scarcity | 0.962224 | 0.324114 | 0.377055 | 0.025553 | 0.177092 | 4.49467 | 5.33537 | 1.65776 | 4.7444 | 3.87374 | 0.904648 | 7.04461 | 29.3508 | 7.24807 | 5.42331 | 1.50746 | 12.477 | 3.74244 | 0.800985 | 9.52863 |
| Water consumption | 1.32593 | 0.30822 | 0.153898 | 0.0374644 | 0.0942856 | 5.87586 | 5.33114 | 5.08538 | 6.05104 | 2.89578 | 0.797573 | 4.71291 | 25.4717 | 5.90153 | 5.40412 | 1.2322 | 19.8344 | 4.97408 | 0.367061 | 4.14542 |

**Supplementary Figure 9.** Relative environmental impacts of semi-automated workflow in real-life at Le Mans laboratory is analysed using the 18 indicators of Product Environmental Footprint method and distributed between reagents, consumables, instruments and human resources used.

Relative impact on workflow (%)

|  | Automated urine particle analyser | UF-Cellsheath | UF-Cellpack SF | UF-Cellpack CR | Fluorocell SF | Fluorocell CF | Rinsing water | Automated plate streaker | Metallic loop | Loop cleaning liquid | Large sponge | Small sponge | Trapezoidal sponges | Medium sponge | Chromogenic agar plate | Automated incubator | Automated antibiogram system | Polystyrene tubes | Distilled water refill | Plastic pipette |
| --- | --- | --- | --- | --- | --- | --- | --- | --- | --- | --- | --- | --- | --- | --- | --- | --- | --- | --- | --- | --- |
| Global warming | 0.914343 | 3.97504 | 0.0401605 | 0.0328468 | 0.00526507 | 0.00493296 | 12.8644 | 3.36341 | 0.00332296 | 1.65421 | 0.00365718 | 0.00102936 | 0.159395 | 0.00254354 | 12.3781 | 0.560059 | 15.3574 | 3.28527 | 0.867385 | 1.19067 |
| Stratospheric ozone depletion | 0.9974 | 4.34443 | 0.047033 | 0.0400691 | 0.00420013 | 0.00402537 | 12.6127 | 3.77009 | 0.00329384 | 3.66809 | 0.00214014 | 0.000733533 | 0.260252 | 0.00208165 | 6.06017 | 1.12572 | 29.3661 | 1.08943 | 0.648959 | 0.913464 |
| Ionizing radiation | 0.17089 | 0.254983 | 0.00272142 | 0.00196767 | 0.000236795 | 0.000383641 | 0.737776 | 1.31912 | 0.000411823 | 0.0972051 | 7.07142e-05 | 3.87106e-05 | 0.1109 | 0.000146437 | 0.298075 | 3.604 | 88.9527 | 0.0518437 | 0.0301673 | 0.0563506 |
| Ozone formation, Human health | 1.13483 | 9.81326 | 0.108433 | 0.102502 | 0.0074252 | 0.00701163 | 13.4441 | 4.15129 | 0.00292619 | 1.49243 | 0.00302334 | 0.000863789 | 0.281868 | 0.00217801 | 6.6992 | 0.571485 | 16.1755 | 1.90284 | 0.376026 | 1.11732 |
| Fine particulate matter formation | 1.5076 | 6.69441 | 0.0729015 | 0.0622875 | 0.00617019 | 0.00555577 | 9.85366 | 5.50616 | 0.00234241 | 1.40739 | 0.00324869 | 0.000817808 | 0.231833 | 0.00189985 | 5.98355 | 0.714309 | 20.3972 | 1.66848 | 0.350857 | 0.869327 |
| Ozone formation, Terrestrial ecosystems | 1.12115 | 9.70533 | 0.107536 | 0.10165 | 0.0074475 | 0.00704387 | 13.6314 | 4.10128 | 0.00296849 | 1.59908 | 0.00310269 | 0.000888019 | 0.281319 | 0.00223867 | 6.78296 | 0.56457 | 15.9802 | 1.93318 | 0.3749 | 1.13639 |
| Terrestrial acidification | 1.19026 | 8.03005 | 0.0867952 | 0.0767617 | 0.00634199 | 0.00598762 | 10.4435 | 4.37898 | 0.00255201 | 1.47833 | 0.00352072 | 0.000881238 | 0.238138 | 0.0020343 | 6.89848 | 0.725259 | 20.071 | 2.03425 | 0.348932 | 0.918493 |
| Freshwater eutrophication | 2.28543 | 3.04122 | 0.0254731 | 0.0202937 | 0.00309188 | 0.00291066 | 10.2264 | 8.26641 | 0.00217 | 1.0815 | 0.00166747 | 0.000507901 | 0.293413 | 0.0013688 | 7.18282 | 0.673383 | 20.8744 | 1.37938 | 0.724239 | 0.694448 |
| Marine eutrophication | 0.797782 | 24.4729 | 0.0158131 | 0.0128415 | 0.0015757 | 0.00151434 | 11.7077 | 3.03912 | 0.00135249 | 11.5167 | 0.00763808 | 0.00124041 | 0.218513 | 0.00183868 | 2.71382 | 1.01445 | 26.4261 | 0.55168 | 0.264831 | 0.30282 |
| Terrestrial ecotoxicity | 0.712985 | 5.14921 | 0.0523285 | 0.0464958 | 0.00401889 | 0.00390562 | 23.011 | 2.78393 | 0.00244744 | 1.28021 | 0.00137162 | 0.000539906 | 0.206121 | 0.00159348 | 3.9144 | 1.25365 | 32.0719 | 0.809514 | 0.370554 | 1.28183 |
| Freshwater ecotoxicity | 3.01976 | 1.03482 | 0.0155966 | 0.0139166 | 0.00264028 | 0.00260577 | 4.45931 | 11.0282 | 0.00278412 | 0.814864 | 0.001425 | 0.000569814 | 0.0458842 | 0.00170207 | 1.50485 | 1.45554 | 40.7653 | 0.290851 | 0.169002 | 0.231838 |
| Marine ecotoxicity | 3.06112 | 1.07971 | 0.016148 | 0.0144488 | 0.00267181 | 0.00263574 | 4.71671 | 11.1664 | 0.00281073 | 0.758726 | 0.00143464 | 0.000574228 | 0.0475659 | 0.00171582 | 1.57038 | 1.40859 | 39.7244 | 0.304588 | 0.174989 | 0.246603 |
| Human carcinogenic toxicity | 1.12139 | 2.45222 | 0.021869 | 0.0179196 | 0.00305321 | 0.00292269 | 6.84395 | 4.13497 | 0.0034044 | 0.911664 | 0.00201058 | 0.00058189 | 0.152642 | 0.00147934 | 10.8303 | 0.729248 | 20.0042 | 2.10576 | 1.07543 | 0.912395 |
| Human non-carcinogenic toxicity | 3.3116 | 1.48023 | 0.0217285 | 0.019011 | 0.0035081 | 0.00342476 | 6.53898 | 11.9812 | 0.00353067 | 0.994232 | 0.00181296 | 0.000729081 | 0.0762737 | 0.0021816 | 2.28741 | 0.994946 | 30.6415 | 0.447651 | 0.249892 | 0.360634 |
| Land use | 0.458138 | 5.97188 | 0.0741131 | 0.0721105 | 0.0143199 | 0.0139101 | 26.3231 | 1.69962 | 0.0248564 | 9.65139 | 0.000455384 | 0.00117523 | 7.29219 | 0.00606402 | 9.20461 | 0.350753 | 9.48636 | 1.61052 | 0.667294 | 1.73146 |
| Mineral resource scarcity | 3.1814 | 1.18142 | 0.0100658 | 0.00802071 | 0.00147719 | 0.00141297 | 3.32043 | 11.5441 | 0.00138512 | 0.469315 | 0.000469219 | 0.000175845 | 0.153273 | 0.000521016 | 1.04856 | 1.12194 | 33.6511 | 0.206738 | 0.106672 | 0.237813 |
| Fossil resource scarcity | 0.733757 | 4.44775 | 0.0483475 | 0.0417568 | 0.00779288 | 0.00754034 | 20.8152 | 2.70288 | 0.00401487 | 2.12517 | 0.00399413 | 0.00142109 | 0.162189 | 0.00383561 | 10.1927 | 0.464158 | 12.7925 | 3.43784 | 0.43592 | 1.60173 |
| Water consumption | 0.757112 | 3.37706 | 0.0299142 | 0.0216174 | 0.00405079 | 0.0041655 | 7.84342 | 2.99798 | 0.00296325 | 3.97697 | 0.00657752 | 0.00131439 | 0.300891 | 0.00252344 | 8.21209 | 1.53876 | 39.2641 | 2.70051 | 0.346114 | 0.693165 |

**Supplementary Figure 10.** Relative environmental impacts of the fully-automated workflow in real-life at Lyon laboratory is analysed using the 18 indicators of Product Environmental Footprint method and distributed between reagents, consumables, instruments and human resources used.
